# Mitochondrial Genomes Provide Insights into the Phylogeny of Culicomorpha (Insecta: Diptera)

**DOI:** 10.3390/ijms20030747

**Published:** 2019-02-11

**Authors:** Xiao Zhang, Zehui Kang, Shuangmei Ding, Yuyu Wang, Chris Borkent, Toyohei Saigusa, Ding Yang

**Affiliations:** 1 Key Lab of Integrated Crop Pest Management of Shandong Province, College of Plant Health and Medicine, Qingdao Agricultural University, Qingdao 266109, China; xzhang_cn@163.com (X.Z.); kangzehui1987@163.com (Z.K.); 2 Department of Entomology, China Agricultural University, Beijing 100193, China; shuangmeiding@cau.edu.cn; 3 College of Plant Protection, Hebei Agricultural University, Baoding 071001, China; wangyy_amy@126.com; 4 California State Collection of Arthropods, California Department of Food and Agriculture, 3294 Meadowview Road, Sacramento, CA 95832, USA; chris.borkent@gmail.com; 5 Saigusa Institute of Insect Natural History, Clover Heights Ropponmatsu 402, 7-1, Baikoen 2-chome, Fukuoka-shi 810-0035, Japan; toyohei_saigusa@yahoo.co.jp

**Keywords:** Culicomorpha, diptera, phylogeny, mitochondrial genome

## Abstract

Culicomorpha is a monophyletic group containing most bloodsucking lower dipterans, including many important vectors of pathogens. However, the higher-level phylogenetic relationships within Culicomorpha are largely unresolved, with multiple competing hypotheses based on molecular sequence data. Here we sequenced four nearly complete mitochondrial (mt) genomes representing four culicomorph families, and combined these new data with published mt genomes to reconstruct the phylogenetic relationships of all eight extant culicomorph families. We estimated phylogenies using four datasets and three methods. We also used four-cluster likelihood mapping to study potential incongruent topologies supported by the different datasets and phylogenetic questions generated by the previous studies. The results showed that a clade containing Ceratopogonidae, Thaumaleidae and Simuliidae was the sister group to all other Culicomorpha; in another clade, the Dixidae was basal to the remaining four families; Chaoboridae, Corethrellidae and Culicidae formed a monophyletic group and the Chironomidae was the sister group to this clade; Culicidae and Corethrellidae were sister groups in all trees. Our study provides novel mt genome data in Culicomorpha for three new family representatives, and the resulting mt phylogenomic analysis helps to resolve the phylogeny and taxonomy of Culicomorpha.

## 1. Introduction

The infraorder Culicomorpha is a well-supported monophyletic group in lower Diptera and includes eight families: Culicidae (mosquitoes), Corethrellidae (frog-biting midges), Dixidae, Chaoboridae (phantom midges), Ceratopogonidae (biting midges), Chironomidae (midges), Simuliidae (black flies) and Thaumaleidae [1]. This clade contains most of the bloodsucking taxa in Diptera (Culicidae, Corethrellidae, Ceratopogonidae and Simuliidae), some of which are important vectors of pathogens that cause human disease [2]. Culicidae, Chironomidae and Ceratopogonidae are particularly ecologically and morphologically rich groups, and they play important roles in many sectors, such as the medical and economic fields [3,4].

Henning (1973) listed synapomorphies of Culicomorpha both in larvae and adults, in particular characters in the adult head, pedicel, wing vein and sperm pump, and in the larval head and pupal leg sheaths [5]. In Henning’s morphological phylogeny, the Culicomorpha was a strongly-supported group divided into two superfamilies, Culicoidea and Chironomoidea. This taxonomy was widely accepted by most subsequent morphological studies [1,6]. The superfamily Culicoidea contains the families Culicidae, Corethrellidae, Dixidae and Chaoboridae, whereas Chironomoidea contains Ceratopogonidae, Chironomidae, Simuliidae and Thaumaleidae. The family Corethrellidae, which had originally been considered to be a subfamily or tribe of Chaoboridae, was upgraded by Wood and Borkent [1]. They also noticed that larvae of Simuliidae and Culicoidea both have a dorsal mandibular brush, which may be a synapomorphy of these two families. This result was controversial because the two families were defined by a limited number of characters [6]. Other studies indicated that Thaumaleidae should be moved out of Chironomoidea and into either Bibionomorpha or Axymyiomorpha [7,8,9,10]. Saether (2000) reconstructed the culicomorph phylogenetic tree using 81 reconsidered morphological characters [11]. This phylogeny varied in several respects, including that Thaumaleidae or the clade (Thaumaleidae + Nymphomyiidae) was sister group to the remaining culicomorph families; Simuliidae and Chironomidae formed a sister clade to the remaining families of this infraorder, and this clade sometimes included the family Ceratopogonidae. Borkent (2012) proposed a novel phylogenetic arrangement of the families in Culicomorpha based on numbers of previously unknown pupal features and all published synapomorphies from every other stage [12]. He considered the Chironomidae to be the sister group of all other Culicomorpha, and proposed a new superfamily Simulioidea containing Ceratopogonidae, Thaumaleidae and Simuliidae; Culicoidea remained as previously recognized by most taxonomic work. In summary, the arrangements of the families in Culicomorpha, especially in Chironomoidea, varied by the morphological studies.

Many phylogenetic studies of Culicomorpha were based on molecular sequence data, and the results also varied. Several earlier molecular studies examined the culicomorph relationships using a single ribosomal gene [13,14] or combinations of molecular markers [15,16]. These earlier studies in particular had many conflicts with each other. Miller’s phylogenetic analyses demonstrated a sister relationship between Chironomidae and the monophyletic lineage Corethrellidae, Chaoboridae and Culicidae [14]. This hypothesis was not supported by Pawlowski et al. (1996) and the Chironomidae was considered to be the sister group of the rest of seven families in Culicomorpha; the Dixidae traditionally considered as closely related to the branch Corethrellidae, Chaoboridae and Culicidae, was not placed close to them [13]. Besides, they both got some unresolved phylogenetic relationships of representatives in the Culicomorpha such as Dixidae, Simuliidae and Ceratopogonidae. Bertone et al. (2008) and Wiegmann et al. (2011) both got three stable branches of Culicomorpha: (Thaumaleidae + Simuliidae), (Ceratopogonidae + Chironomidae) and the traditionally recognized Culicoidea, but their topological relationships were different [15,16]. In addition, the relationships of Corethrellidae, Chaoboridae and Culicidae were different in these phylogenetic studies. Pawlowski et al. and Wiegmann et al. supported the sister relationship between Corethrellidae and Chaoboridae, while Miller et al. and Bertone et al. considered the close relationship between Chaoboridae and Culicidae [13,14,15,16]. More recent studies have clarified some of those conflicts by using more advanced analytical methods and more molecular markers. Kutty et al. (2018) used whole-transcriptome shotgun phylogenomic approach to clarify the relationships among all families of Culicomorpha [17]. The transcriptomic data presented a well-supported monophyletic superfamily Culicoidea comprising Dixidae + (Corethrellidae + (Chaoboridae + Culicidae)). However, Chironomoidea was not monophyletic, since the clade (Chironomidae + Ceratopogonidae) was placed as the sister group of all remaining Culicomorpha (though with low support). The other clade of Chironomoidea was strongly supported as (Thaumaleidae + Simuliidae) + Culicoidea. 

The phylogeny of Culicomorpha have also been reconstructed using mitochondrial (mt) DNA [18,19]. Beckenbach and Borkent used one fragment of mt DNA, the mt cytochrome oxidase subunit 2 gene (*cox2*), to resolve the phylogeny of Ceratopogonidae [18]. Their result was mostly congruent with former morphological analyses, suggesting Ceratopogonidae and Chironomidae were sister-groups, and that Simuliidae was sister to this clade. In Beckenbach’s recent mt phylogenetic analyses, the complete mt genomes were used, but there were only four complete mt data from species of three families in Culicomorpha [19]. In the past decade, more whole mt genomes have been sequenced and widely used for reconstructing phylogenetic relationships, although there have been some criticisms of the use of mt genomes for phylogenetic analysis, due to the accelerated substitution rate and relatively higher compositional heterogeneity [20,21]. Nevertheless, the mt genome is still a useful marker for understanding the phylogenetic relationships, and has been used in many insect groups, including the Diptera [19,22,23,24,25]. As of September 2018, there were more than four hundred complete or nearly complete culicomorph mt genome sequences available in GenBank. Most of these mt genomes were from species of Culicidae, whereas only eight of them were from species of Ceratopogonidae (1), Dixidae (2), Simuliidae (2) and Chironomidae (3) (Table 1). Unfortunately, there was no available mt genome data from Thaumaleidae, Corethrellidae or Chaoboridae. Because there were nearly 200 mt sequences of Culicidae, many of which represented the same species or genus, we chose 8 mt sequences which belong to the five genera listed in Table 1.

In this study, we sequenced four mt genomes, representing Thaumaleidae, Corethrellidae, Chaoboridae and Simuliidae respectively. The other 16 published mt genomes of culicomorph species were downloaded from the National Center of Biotechnology Information (NCBI). We used four datasets and reconstructed 12 phylogenetic trees based on Bayesian inference (BI) and maximum likelihood (ML) methods to explore relationships among the major groups of Culicomorpha. In particular, we used four-cluster likelihood mapping (FcLM) to study potential incongruent topologies supported by different datasets in this study and phylogenetic questions generated by the previous studies. We focus on the following questions: (1) Are Thaumaleidae and Simuliidae sister groups? (2) Is the traditional Chironomoidea a monophyletic group? (3) Which family is the sister-group of the branch (Chaoboridae + (Corethrellidae + Culicidae)), Dixidae or Chironomidae? (4) What is the relationships of Corethrellidae, Chaoboridae and Culicidae?

## 2. Results

### 2.1. Phylogenetic Analyses

**Figure 1:**
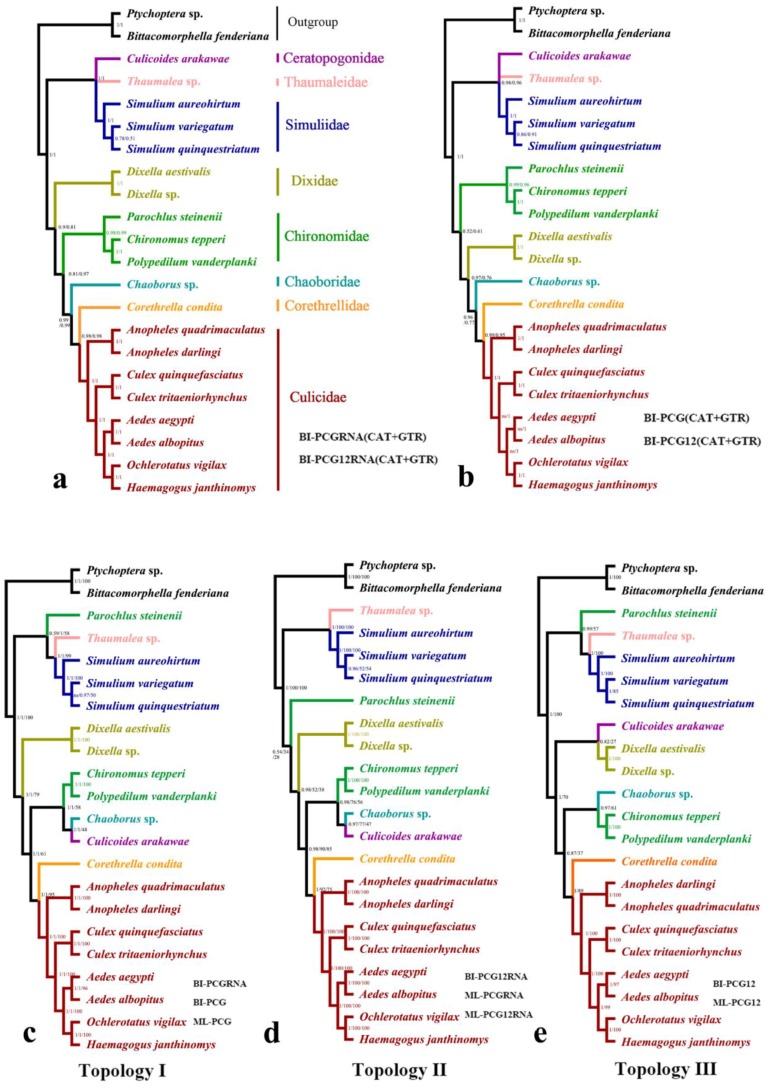
Phylogenetic trees of Culicomorpha based on mt genome data. (**a**) Bayesian inference (BI) tree based on protein-coding gene (PCG) 19 tRNAs and two rRNAs (RNA) and PCG12RNA with heterogeneous models (CAT + GTR); (**b**) BI tree based on PCG and PCG12 with heterogeneous models; (**c**) BI tree based on PCGRNA and PCG, and ML tree based on PCG with homogeneous models; (**d**) BI tree based on PCG 12RNA and ML tree based on PCGRNA and PCG12RNA with homogeneous models; (**e**) BI and ML tree based on PCG12 with homogeneous models. Numbers above the branches are posterior probabilities or bootstrap percentages, ns = not support.

Twenty species from the eight families of Culicomorpha were included in the phylogenetic analysis. Twelve phylogenetic trees were reconstructed based on four datasets using BI and ML methods (Figure 1, Figure S1). Monophyly of the Culicomorpha was well-supported and all families were recovered as monophyletic groups, except for Chironomidae, which was recovered as paraphyletic using the homogeneous model in BI and ML analyses (Figure 1). When we used the heterogeneity model in PhyloBayes (CAT + GTR), the monophyly of Chironomidae was supported (posterior probabilities (PP) > 0.95). However, there were two alternative topologies depending on which dataset was used (Figure 1a,b): in the trees based on PCGRNA and PCG12RNA, the Culicomorpha was recovered as paraphyletic, with Thaumaleidae, Simuliidae and Ceratopogonidae forming one branch (PP = 1) and the other five families assembling together with Dixidae as sister to the other families (PP = 0.9/0.81) (Figure 1a); in the trees based on PCG12 and PCG, the relationships within the latter clade changed to Chironomidae recovered as sister to the other four families with low posterior probabilities (Figure 1b). The BI and ML trees under the homogeneous model had three different topologies (Topology I, II, III) (Figure 1c–e). The BI analysis based on PCGRNA and PCG, as well as the ML analysis based on PCG suggested topology I, in which the *Parochlus steinenii* (a member of Chironomidae) was placed as sister to Thaumaleidae and Simuliidae, thus making Chironomidae a paraphyletic group. Dixidae was recovered as sister to the remaining five families (PP = 1/1 and BP = 79), which formed a well-supported clade as (Chironomidae + (Chaoboridae + Ceratopogonidae)) + (Corethrellidae + Culicidae) (PP = 1/1 and most BP =61) (Figure 1c). Topology II, which was recovered in both the BI analysis based on PCG12RNA and the ML analysis based on both PCGRNA and PCG12RNA, (Figure 1d) was very similar to Topology I. The only difference was the placement of *P. steinenii* (Chironomidae). The BI and ML analyses based on PCG12 consistently supported topology III: *P. steinenii* was recovered as sister to Thaumaleidae and Simuliidae (BP = 0.99 and BP = 57), Ceratopogonidae was recovered as sister to Dixidae (BP = 0.82 and BP = 27), and the other four families assembled together as (Chironomidae + Chaoboridae) + (Corethrellidae + Culicidae) (Figure 1e). Despite these conflicts among topologies I, II and III (Figure 1c–e), there were two stable clades: the sister relationships between 1) Thaumaleidae and Simuliidae and 2) Corethrellidae and Culicidae (PP > 0.95 and BP > 79).

### 2.2. FcLM Analysis

**Figure 2:**
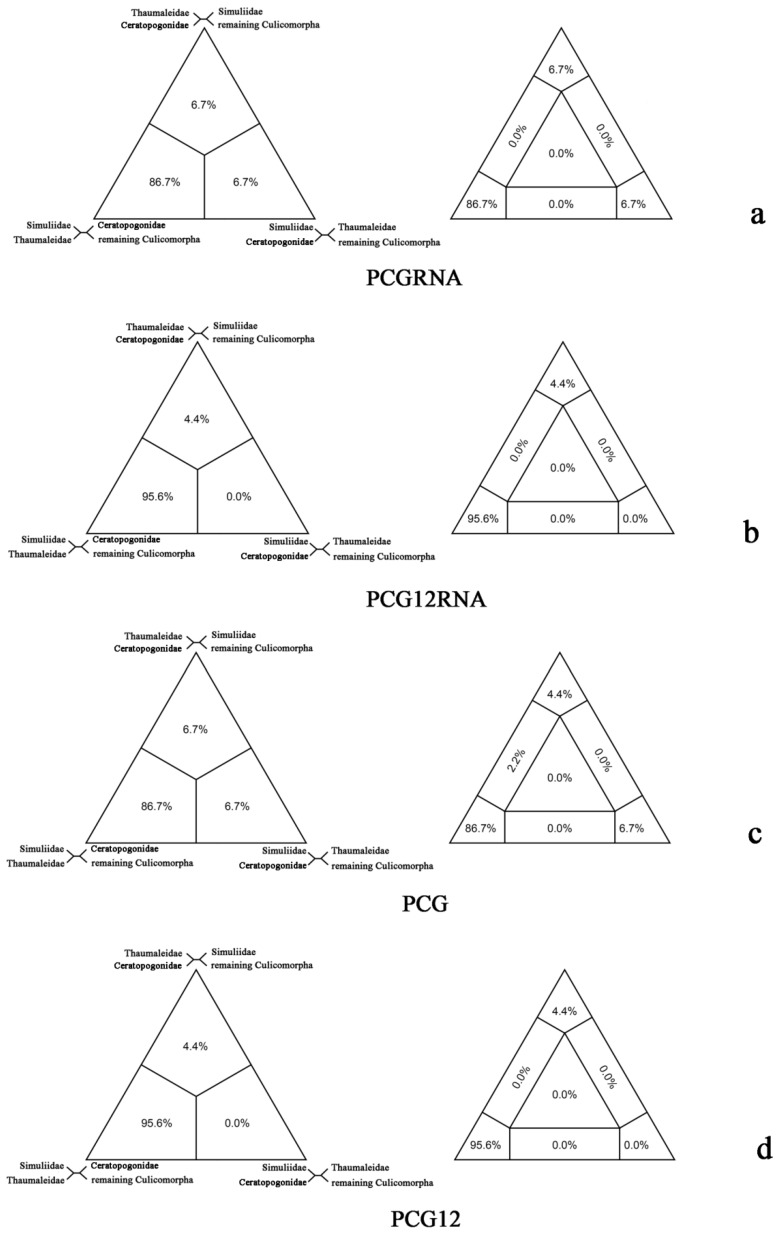
Results of Four-cluster Likelihood Mapping as 2D simplex graphs. (**a**) Four-cluster Likelihood Mapping based on PCGRNA; (**b**) four-cluster Likelihood Mapping based on PCG12RNA; (**c**) four-cluster Likelihood Mapping based on PCG; (**d**) four-cluster Likelihood Mapping based on PCG12.

The FcLM analysis showed a support for the sister-group relationship between Thaumaleidae and Simuliidae (86.7%/95.6%/86.7%/95.6%) (Figure 2). Alternative relationships were weakly supported: Ceratopogonidae as sister group to Thaumaleidae (6.7%/4.4%/6.7%/4.4%), and Ceratopogonidae as sister group to Simuliidae (6.7%/0.0%/6.7%/0.0%). The results were concordant with our BI trees based on PCGRNA and PCG12RNA datasets using a heterogeneous model (Figure 1a).

**Figure 3:**
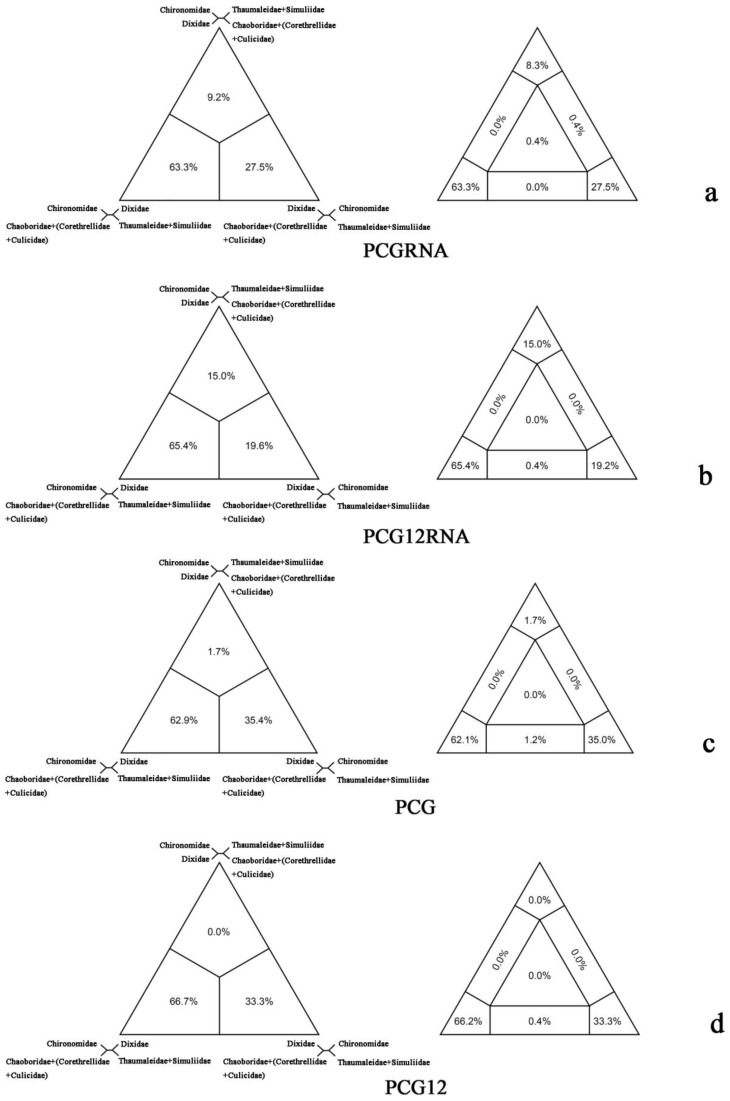
Results of Four-cluster Likelihood Mapping as 2D simplex graphs. (**a**) Four-cluster Likelihood Mapping based on PCGRNA; (**b**) four-cluster Likelihood Mapping based on PCG12RNA; (**c**) four-cluster Likelihood Mapping based on PCG; (**d**) four-cluster Likelihood Mapping based on PCG12.

The FcLM analysis also showed a clear preference for the sister-group relationship between Chironomidae and (Chaoboridae + (Corethrellidae + Culicidae)) (63.3%/65.4%/62.9%/66.7%) (Figure 3). Alternative relationships were weakly supported: Dixidae as sister group to (Chaoboridae + (Corethrellidae + Culicidae)) (27.5%/19.6%/35.4%/33.3%), and (Thaumaleidae + Simuliidae) as sister group to (Chaoboridae + (Corethrellidae + Culicidae)) (9.2%/15.0%/1.7%/0%). The results were compatible with our BI trees based on PCGRNA and PCG12RNA datasets using a heterogeneous model (Figure 1a).

**Figure 4:**
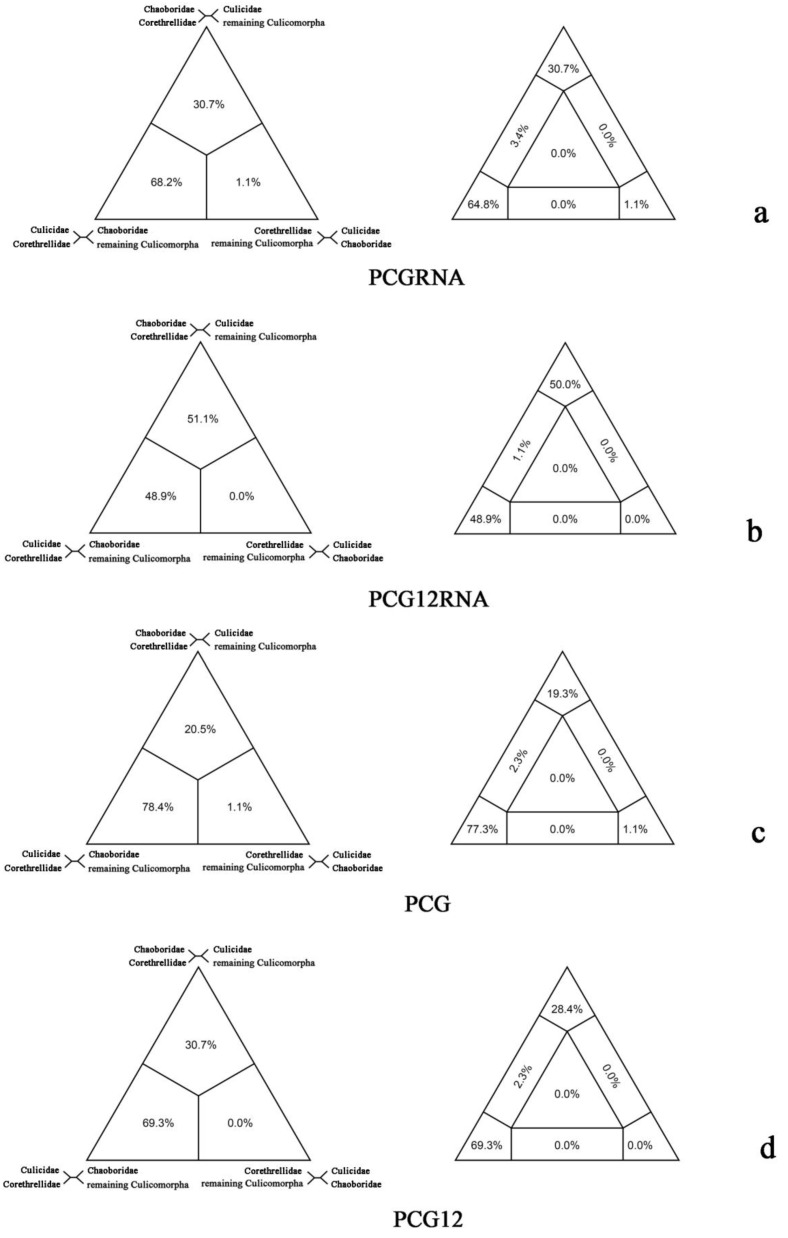
Results of Four-cluster Likelihood Mapping as 2D simplex graphs. (**a**) Four-cluster Likelihood Mapping based on PCGRNA; (**b**) four-cluster Likelihood Mapping based on PCG12RNA; (**c**) four-cluster Likelihood Mapping based on PCG; (**d**) four-cluster Likelihood Mapping based on PCG12.

About the relationships between Chaoboridae, Corethrellidae and Culicidae, the FcLM analysis showed an obvious preference for the sister-group relationship between Culicidae and Corethrellidae (68.2%/48.9%/78.4%/69.3%) (Figure 4). Alternative relationships were weakly supported: Chaoboridae as sister group to Corethrellidae (30.7%/51.1%/20.5%/30.7%), and Chaoboridae as sister group to Culicidae (1.1%/0.0%/1.1%/0.0%). The results were in agreement with all phylogenetic trees based on four datasets (Figure 1).

## 3. Discussion

In this study we applied a variety of strategies to explore the phylogenetic relationships of the culicomorph insects using mt genome sequences. In both BI (MrBayes) and ML trees, the position of Ceratopogonidae was unstable and the monophyly of Chironomidae was not supported. Some studies have suggested erroneous phylogenetic reconstruction may be attributed to artificial bias (e.g., long branch attraction, LBA) [26]. Here, we used heterogeneous models (CAT + GTR) to reduce the effects of LBA. Using this approach, the Chironomidae was recovered as a monophyletic group. However, the analyses with heterogeneous models did not provide a stable position of Chironomidae within Culicomorpha. The relationship of Ceratopogonidae, Thaumaleidae and Simuliidae was also unclear in the trees estimated under heterogeneous models. The FcLM analysis was used to solve these problems and the results supported the positions of Chironomidae as showed in phylogenetic trees based on PCGRNA and PCG12RNA with heterogeneous models (Figure 1a) and the sister-group relationship between Thaumaleidae and Simuliidae.

Although the heterogeneous models could effectively reduce the effects of LBA, the longest branch (Ceratopogonidae) still had negative effects on the topologies which was mainly manifested in the unclear relationships between Ceratopogonidae, Thaumaleidae and Simuliidae (Figure 1a,b). In order to check whether the Ceratopogonidae had an obvious effect on topologies, we used the same methods to reconstruct additional twelve phylogenetic trees with all Ceratopogonidae removed (Figure S2). Most topologies had no obvious differences after removing the Ceratopogonidae, with the only exception of the phylogenetic tree based on PCGRNA with heterogeneous models, where instead of the Chironomidae, the Dixidae was sister group to (Chaoboridae + (Corethrellidae + Culicidae)). These two alternative topologies were compared in the previous section and have been analyzed in FcLM studies which supported the Chironomidae as sister group to (Chaoboridae + (Corethrellidae + Culicidae)). In summary, LBA had no obvious effect on the topologies estimated under heterogeneous models.

**Figure 5:**
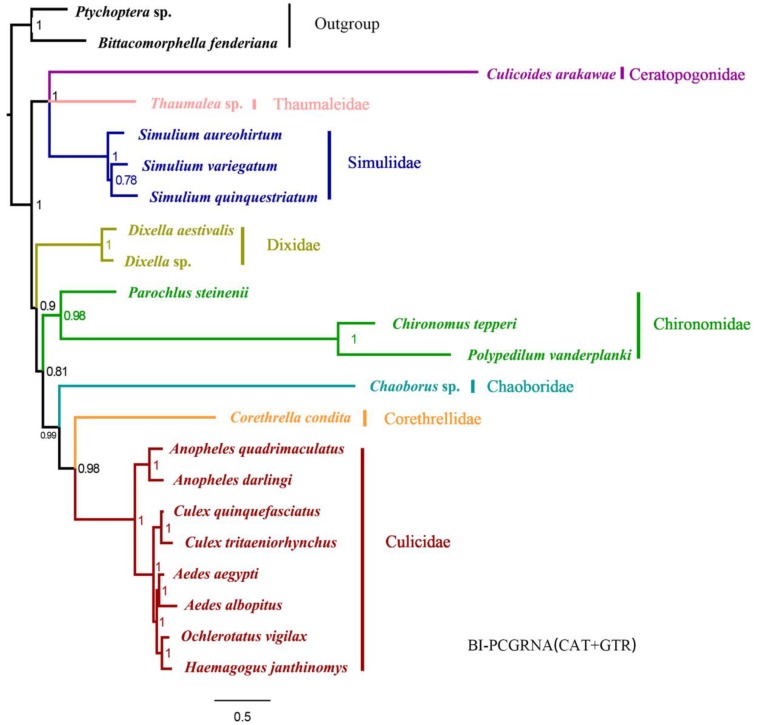
Phylogenetic tree of Culicomorpha based on PCGRNA with heterogeneous models. Phylogram of relationships resulting from BI with *Bittacomorphella fenderiana* and *Ptychopter* sp. as outgroups. Numbers above the branches are posterior probabilities.

The results from our study have some major implications for the taxonomy and higher-level relationships of Culicomorpha. We recovered two main lineages of Culicomorpha: a clade of (Ceratopogonidae + Thaumaleidae + Simuliidae) and a clade of the remaining Culicomorpha. Therefore, a modified Culicoidea contains five families: Dixidae + (Chironomidae + (Chaoboridae + (Corethrellidae + Culicidae))) (Figure 5).

Although traditional morphologic hypotheses did not support the close relationship between Thaumaleidae and Simuliidae, molecular data provided consistent support for this relationship [13,16,27]. As suggested by Pawlowski et al., two certain clades: (Thaumaleidae, Simuliidae) and (Chaoboridae, Corethrellidae and Culicidae), were confirmed [13]. This proposal was based on molecular evidence, and also on shared morphological features of Thaumaleidae and Simuliidae that differ from other culicomorphans. As proposed by Bertone et al., adults in these two families were particularly robust compared with the delicate, midge-like forms of other culicomorph insects [16]. The close relationship of Thaumaleidae and Simuliidae was strongly supported by some synapomorphies that were found in each life stage of these two families. Adults of these two families both have short and stout antennae that are not modified in the males. However, the synapomorphy was considered to be lost in these two families by Wood & Borkent [1]. Because Thaumaleidae and Simuliidae were sister taxa according to most of our analyses based on mt genome data, we accepted that the characters above may therefore be synapomorphies for Thaumaleidae and Simuliidae.

In Henning’s (1973) hypothesis, Ceratopogonidae was a member of the superfamily Chironomoidea and was sister to Chironomidae [5]. This relationship was supported by some subsequent studies [1,6,15]. However, Ceratopogonidae, together with Thaumaleidae and Simuliidae, comprised the superfamily Simulioidea in Borkent’s analysis, based on six pupal and one adult synapomorphies [12]. Our results were compatible with Borkent’s analyses, although the sister-group relationship between Ceratopogonidae and (Thaumaleidae + Simuliidae) was not consistently supported in our BI trees based on heterogeneous models (CAT + GTR). However, the FcLM analysis gave us an obvious support for the sister-group relationship between Thaumaleidae and Simuliidae (rather than Ceratopogonidae and Thaumaleidae or Ceratopogonidae and Simuliidae). The Chironomidae, which was traditionally recognized as sister group of Ceratopogonidae, was arranged in the Culicoidea. In our analyses, the Chironomidae was recognized as sister to (Chaoboridae + (Corethrellidae + Culicidae)), which was coincident with Miller’s phylogenetic analyses. The Dixidae was the sister group of the above branch. These arrangements were challenging because the familial composition and the relationship of Culicoidea in previous studies seemed stable. However, mt genomes provided a newly insight into the phylogeny of this clade. Accordingly, the traditional Chironomoidea, which was a problematic taxon, was recognized as a paraphyletic group. However, Ceratopogonidae and Chironomidae both contain thousands of described species while there are only four mt data from species of these families. Because the low sampling density of these two families might be bring errors into the phylogenetic reconstruction of these clades, a broader taxonomic sample is needed for future studies.

Consistent with other studies based on molecular data, our mt data did not support the relationships among the Chaoboridae, Corethrellidae and Culicidae presented by Wood & Borkent (1989) and Oosterbroek & Courtney (1995). In these previous analyses, the Chaoboridae was strongly support as the sister group of the Culicidae. Corethrellidae, which had been considered either a tribe or subfamily within the Chaoboridae, was upgraded to a new family according to their precocious development of the adult eye within the larva and the presence of a pair of movable lobes or paddles at the apex of the pupal abdomen, as synapomorphies of Culicidae and Chaoboridae [1]. However, there have been some disagreements about the relationships among Corethrellidae, Culicidae and Chaoboridae by molecular evidence [13,15]. They supported the close relationship between Chaoboridae and Corethrellidae, but the morphological synapomorphies were not reported. All our results strongly supported the sister relationship between Corethrellidae and Culicidae. This may be a new viewpoint for this clade. Adults Corethrellidae and Culicidae both feed on the blood of vertebrate, and the wing venations of them are very similar. More taxonomic studies on these families and multiple phylogenetic analysis methods are needed to help us understand the relationship of this clade in further studies.

## 4. Materials and Methods

### 4.1. Taxon Sampling

Nucleotide sequences of mt genomes for 18 culicomorph insects were obtained from the NCBI. We also sequenced four new mt genome sequences, *Chaoborus* sp., *Corethrella condita*, *Simulium quinquestriatum* and *Thaumalea* sp. (GenBank accession number: MK281356–MK281359), representing four culicomorph families (Chaoboridae, Corethrellidae, Simuliidae and Thaumaleidae). Collecting information for the specimens was presented in Table S1. Specimens were preserved in 95% ethanol immediately after being collected in the field, and then stored in −20 °C freezers at China Agricultural University (CAU).

### 4.2. DNA Extraction, PCR and Sequencing

We extracted DNA from muscle tissues of individual specimens using the TIANamp Genomic DNA Kit (TIANGEN, Beijing, China). The mt DNA fragments were amplified using standard primers for insects [28] and the nonconservative sequences were amplified using primers designed based on these known nucleotide fragments (Table S2). Polymerase Chain Reaction (PCR) amplification conditions contain a hot-start denaturation step at 95 °C for 30 s, 40 cycles of denaturation at 95 °C for 10 s, annealing at 40–55 °C for 50 s, extension at 65 °C for 1 kb/min and a final elongation step at 65 °C for 10 min. NEB Long Taq DNA polymerase (New England Biolabs, Ipswich, MA, USA) was used in PCR amplification and electrophoresis in a 1% agarose gel stained with GoldView nucleic acid dye was used to evaluate the quality of PCR products. Amplified products were sequenced in both strands using the BigDye Terminator Sequencing Kit (Applied Bio Systems, Perkin Elmer, Foster City, CA, USA) and an ABI 3730XL Genetic Analyzer (PE Applied Biosystems, San Francisco, CA, USA).

### 4.3. Sequence Annotation

All sequences were annotated manually following the method proposed by Cameron [29]. Sequences were identified and aligned using BioEdit 7.0.5.3 [30] and assembled using DNAMAN v5.2.2 [31]. The transfer RNA (tRNA) genes were initially identified with tRNAscan-SE v2.0 using invertebrate mt predictors with a cutoff score of 1, or by sequence similarity to tRNAs of other Culicomorpha [32]. The ribosomal RNA (rRNA) genes were detected by alignment with homologous sequences obtained from the published culicomorph species. The boundaries of protein-coding genes (PCGs) were identified based on open reading frames provided by ORF Finder (https://www.ncbi.nlm.nih.gov/gorf/gorf.html), and then checked manually by aligning with homologous sequences using MEGA 5.0 [31]. The organization, nucleotide composition and codon usage of four Culicomorpha mt genomes were provided (Tables S3–S5).

### 4.4. Dataset Concatenation and Phylogenetic Analysis

We used a total of 20 culicomorph species as ingroup taxa and two outgroup taxa, *Bittacomorphella fenderiana* and *Ptychopter* sp. (Ptychopteridae), for phylogenetic analysis (Table 1). Because we failed to get the complete mt genome sequences of *Chaoborus* sp., *Corethrella condita*, *Thaumalea* sp. and *Simulium quinquestriatum*, three tRNA genes (*tRNA^Ile^*, *tRNA^Gln^* and *tRNA^Met^*) and partial 12S rRNA were omitted from the datasets. Individual genes were aligned in MEGA 5.0 and concatenated in SequeceMatrix v1.7.8 [33]. Four data matrices were generated for phylogenetic analyses: (1) the 13 PCGs, two rRNAs and 19 tRNAs (PCGRNA, 14,279 bp); (2) the first and second codon positions of the 13 PCGs, 19 tRNAs and two rRNAs (PCG12RNA, 10,611 bp); (3) the 13 PCGs (PCG, 11,004 bp); and (4) the first and second codon positions of the 13 PCGs (PCG12, 7,336 bp).

BI and ML methods were used for phylogenetic analyses. BI analyses were conducted using MrBayes v3.2.2 [34] and PhyloBayes [35]. ML analysis was conducted using RAxML-HPC2 v8.1.11 [36]. The best-fit partitioning scheme and the substitution models for each partition were determined using PartitionFinder v1.1.1 [37] under BIC (Table S6). The BI analysis in MrBayes was performed using GTR + I + G and HKY+I+G models and two simultaneous runs of 5-10 million generations for each dataset. Convergence of the BI runs (the standard deviation of split frequencies < 0.01) was tested using the program Are We There Yet (AWTY) [38]. The BI analysis in PhyloBayes was conducted under the heterogeneous model CAT-GTR (maxdiff less than 0.3). ML analysis was conducted with GTR+I+G and HKY+I+G models, and the reliability of the inferred topology was assessed by performing 500 rapid bootstrap replicates.

The program TreePuzzle v5.3 [39,40] was used for FcLM analysis to evaluate single phylogenetic splits. FcLM testing was used to evaluate three phylogenetic questions: What are the relationships of Ceratopogonidae, Thaumaleidae and Simuliidae? Whether Dixidae or Chironomidae is the sister group of the clade (Chaoboridae + (Corethrellidae + Culicidae))? What are the relationships of Corethrellidae, Chaoboridae and Culicidae? (Table 2). For these test, all species in the dataset were grouped into four clusters representing alternative resolutions of the phylogenetic hypothesis in question.

## Figures and Tables

**Table 1:** Summary of taxonomic groups used in this study.

Infraorders	Family	Subfamily	Species	GenBank
Culicomorpha	Culicidae	Anophelinae	*Anopheles darlingi*	NC_014275
			*Anopheles quadrimaculatus*	NC_000875
		Culicinae	*Aedes albopitus*	NC_006817
			*Aedes aegypti*	NC_035159
			*Culex quinquefasciatus*	NC_014574
			*Culex tritaeniorhynchus*	NC_028616
			*Haemagogus janthinomys*	NC_028025.1
			*Ochlerotatus vigilax*	NC_027494
	Chironomidae	Chironominae	*Chironomus tepperi*	NC_016167
			*Polypedilum vanderplanki*	NC_028015.1
		Podonominae	*Parochlus steinenii*	KT003702
	Simuliidae	Simuliinae	*Simulium quinquestriatum* *	MK281358
			*Simulium aureohirtum*	KP793690.1
			*Simulium variegatum*	NC_033348.1
	Dixidae	–	*Dixella aestivalis*	NC_029354.1
		–	*Dixella* sp.	KM245574
	Ceratopogonidae	Ceratopogoninae	*Culicoides arakawae*	NC_009809
	Chaoboridae	Chaoborinae	*Chaoborus* sp. *	MK281356
	Corethrellidae	–	Corethrella condita*	MK281357
	Thaumaleidae	–	*Thaumalea* sp. *	MK281359
Psychodomorpha	Ptychopteridae	Ptychopterinae	*Ptychoptera* sp.	NC_016201
		Bittacomorphinae	*Bittacomorphella fenderiana*	JN_861745

* Species newly sequenced for phylogenetic analysis in this study.

**Table 2:** The three datasets designed to evaluate three phylogenetic questions of Culicomorpha.

Phylogenetic Questions	Groups	Number of Species
What is the relationships of Ceratopogonidae, Thaumaleidae and Simuliidae?	G1(a): Ceratopogonidae	1
	G2(b): Thaumaleidae	1
	G3(c): Simuliidae	3
	G4(d): remaining Culicomorpha	15
Which family is the sister-group of the branch (Chaoboridae + (Corethrellidae + Culicidae)), Dixidae or Chironomidae?	G1(a): Dixidae	2
	G2(b): Chironomidae	3
	G3(c): Chaoboridae + (Corethrellidae + Culicidae)	10
	G4(d): Thaumaleidae + Simuliidae	4
What is the relationships of Corethrellidae, Chaoboridae and Culicidae?	G1(a): Chaoboridae	1
	G2(b): Corethrellidae	1
	G3(c): Culicidae	8
	G4(d): remaining Culicomorpha	10

## Supplementary Materials

Supplementary materials can be found at http://www.mdpi.com/1422-0067/20/3/747/s1. Table S1: Collection information of specimens. Table S2: Primers used in this study. Table S3: Organization of four Culicomorpha mt genomes. Table S4: Nucleotide composition of four Culicomorpha mt genomes. Table S5: Codon usage of four Culicomorpha mt genomes. Table S6: The best partitioning scheme selected by PartitionFinder for different datasets. Figure S1: BI and ML trees based on PCGRNA, PCG12RNA, PCG and PCG12. Figure S2: BI and ML trees based on PCGRNA, PCG12RNA, PCG and PCG12 with removing the Ceratopogonidae.
