# Supplementary material for: Mitochondrial Genomes Provide Insights into the Phylogeny of Culicomorpha (Insecta: Diptera)
Source: Int J Mol Sci. 2019 Feb 11;20(3):747. doi: 10.3390/ijms20030747 (PMC6387087; doi:10.3390/ijms20030747)
Supplement: Supplementary file 1 [file ijms-20-00747-s001.zip › Supplementary Files/Figure S1.pdf]

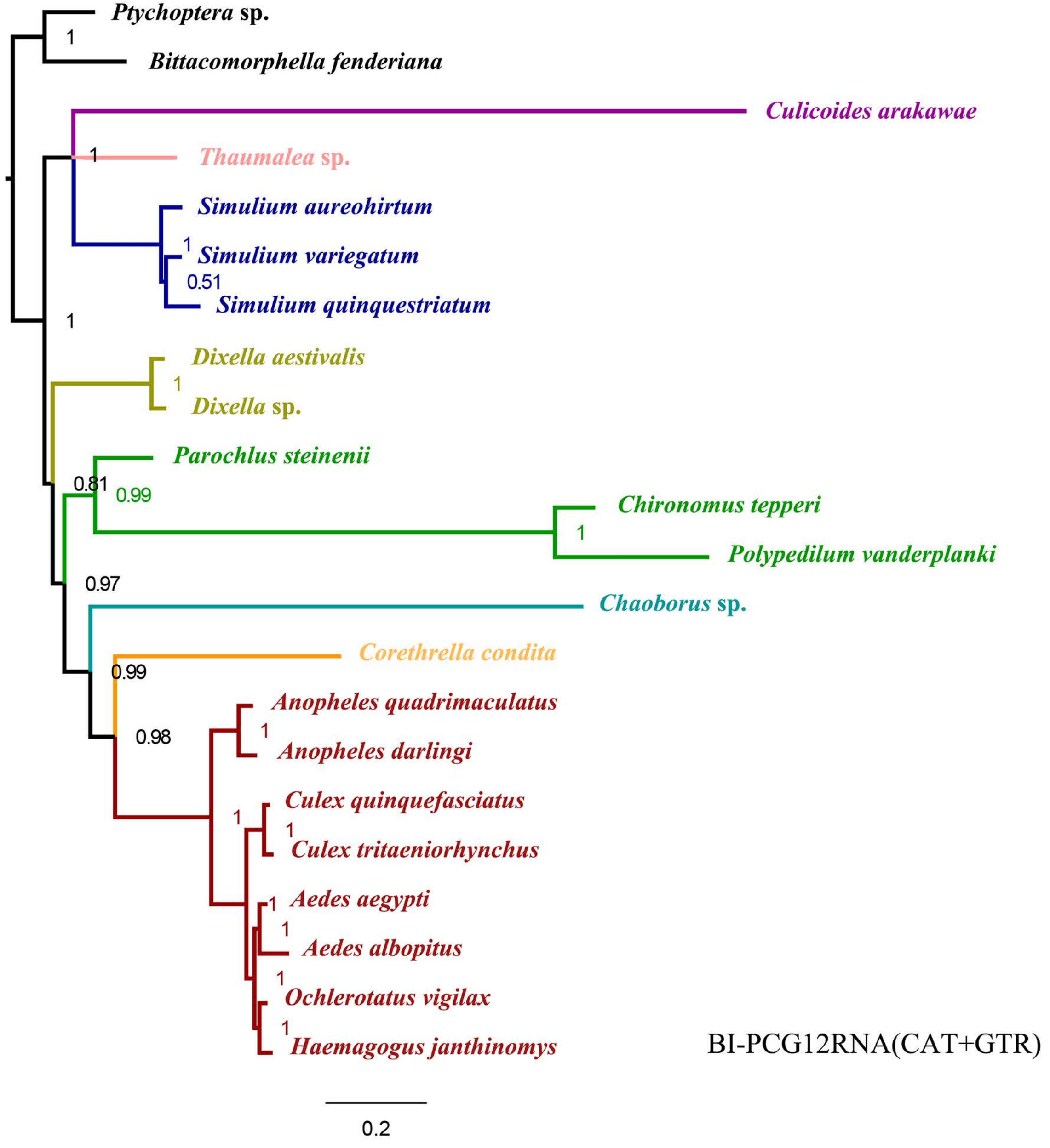

Ptychoptera sp.
Bittacomorphella fenderiana
Culicoides arakawae
Thaumalea sp.
Simulium aureohirtum
Simulium variegatum
Simulium quinquestriatum
Dixella aestivalis
Dixella sp.
Parochlus steinenii
Chironomus tepperi
Polypedilum vanderplanki
Chaoborus sp.
Corethrella condita
Anopheles quadrimaculatus
Anopheles darlingi
Culex quinquefasciatus
Culex tritaeniorhynchus
Aedes aegypti
Aedes albopitus
Ochlerotatus vigilax
Haemagogus janthinomys
1
1
1
0.51
1
1
0.81
0.99
1
0.97
0.99
0.98
1
1
1
1
1
1
1
BI-PCG12RNA(CAT+GTR)
0.2

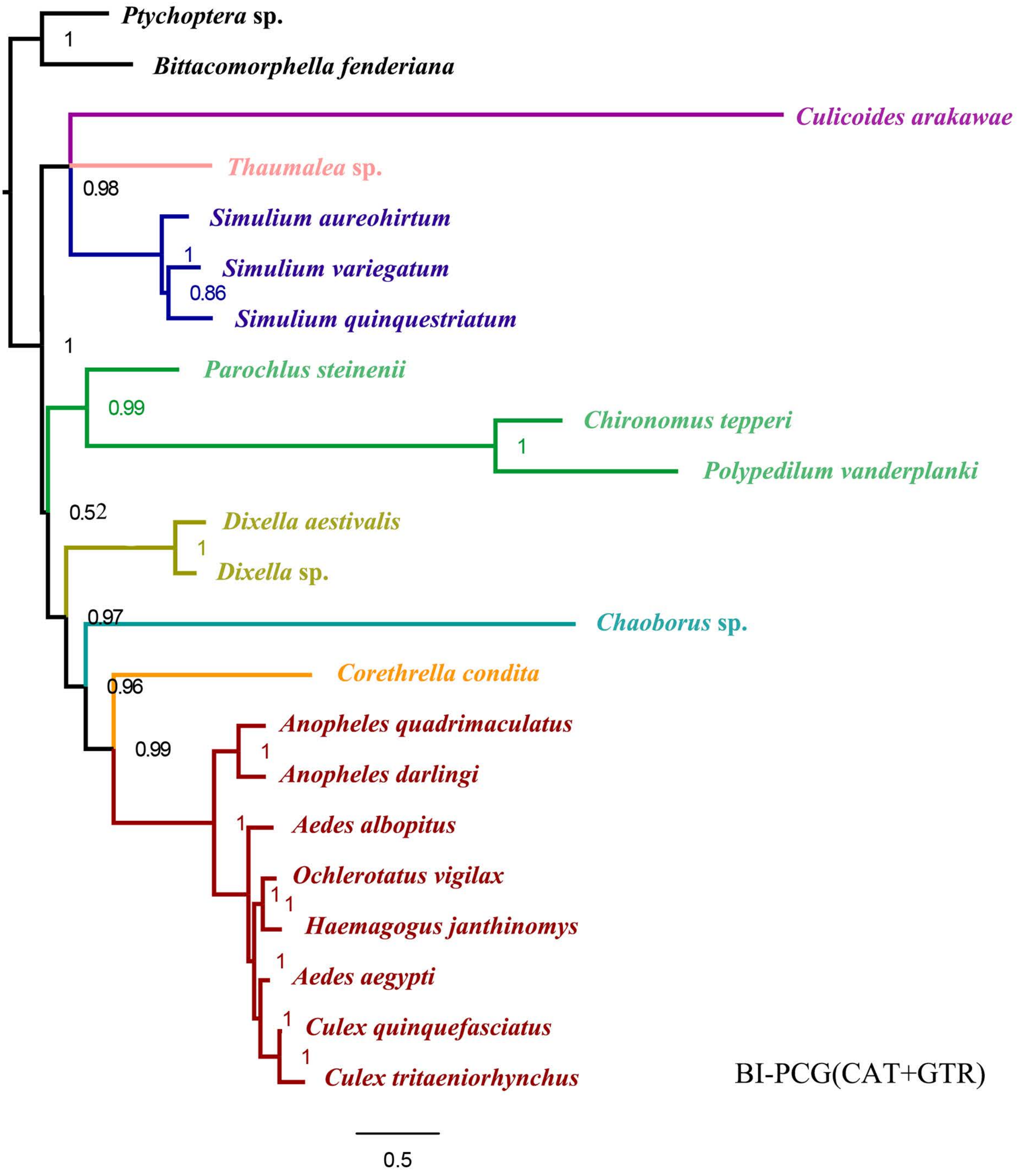
Ptychoptera sp.
Bittacomorphella fenderiana
Culicoides arakawae
Thaumalea sp.
Simulium aureohirtum
Simulium variegatum
Simulium quinquestriatum
Parochlus steinenii
Chironomus tepperi
Polypedilum vanderplanki
Dixella aestivalis
Dixella sp.
Chaoborus sp.
Corethrella condita
Anopheles quadrimaculatus
Anopheles darlingi
Aedes albopitus
Ochlerotatus vigilax
Haemagogus janthinomys
Aedes aegypti
Culex quinquefasciatus
Culex tritaeniorhynchus
1
0.98
1
0.86
1
0.99
1
0.52
1
0.97
0.96
0.99
1
1
1
1
1
1
1
BI-PCG(CAT+GTR)
0.5

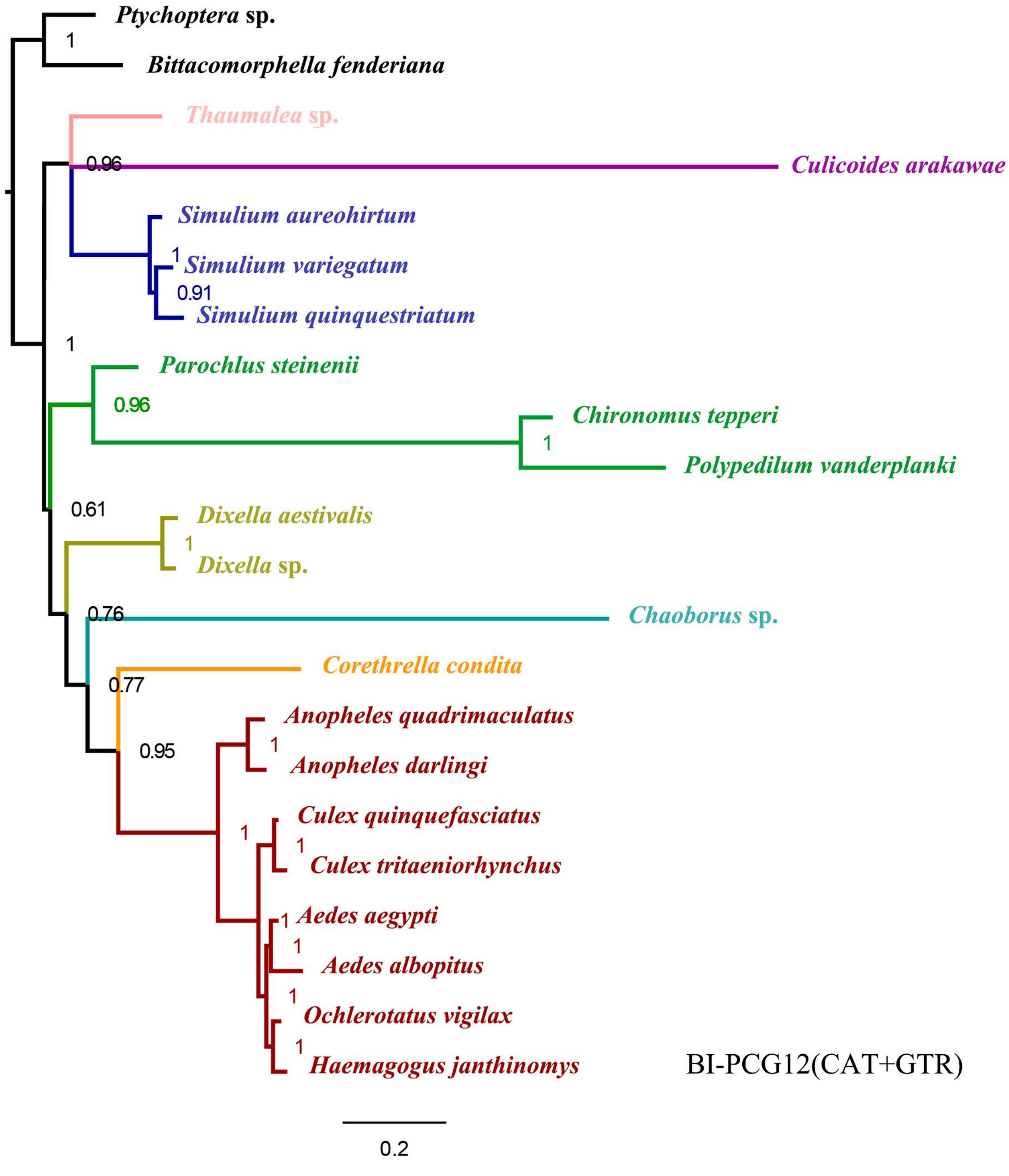

Ptychoptera sp.
1
Bittacomorphella fenderiana
Thaumalea sp.
0.96
Culicoides arakawae
Simulium aureohirtum
1
Simulium variegatum
0.91
Simulium quinquestriatum
1
Parochlus steinenii
0.96
Chironomus tepperi
1
Polypedilum vanderplanki
0.61
Dixella aestivalis
1
Dixella sp.
0.76
Chaoborus sp.
Corethrella condita
0.77
Anopheles quadrimaculatus
1
0.95
Anopheles darlingi
Culex quinquefasciatus
1
1
Culex tritaeniorhynchus
Aedes aegypti
1
1
Aedes albopitus
1
Ochlerotatus vigilax
1
Haemagogus janthinomys
BI-PCG12(CAT+GTR)
0.2

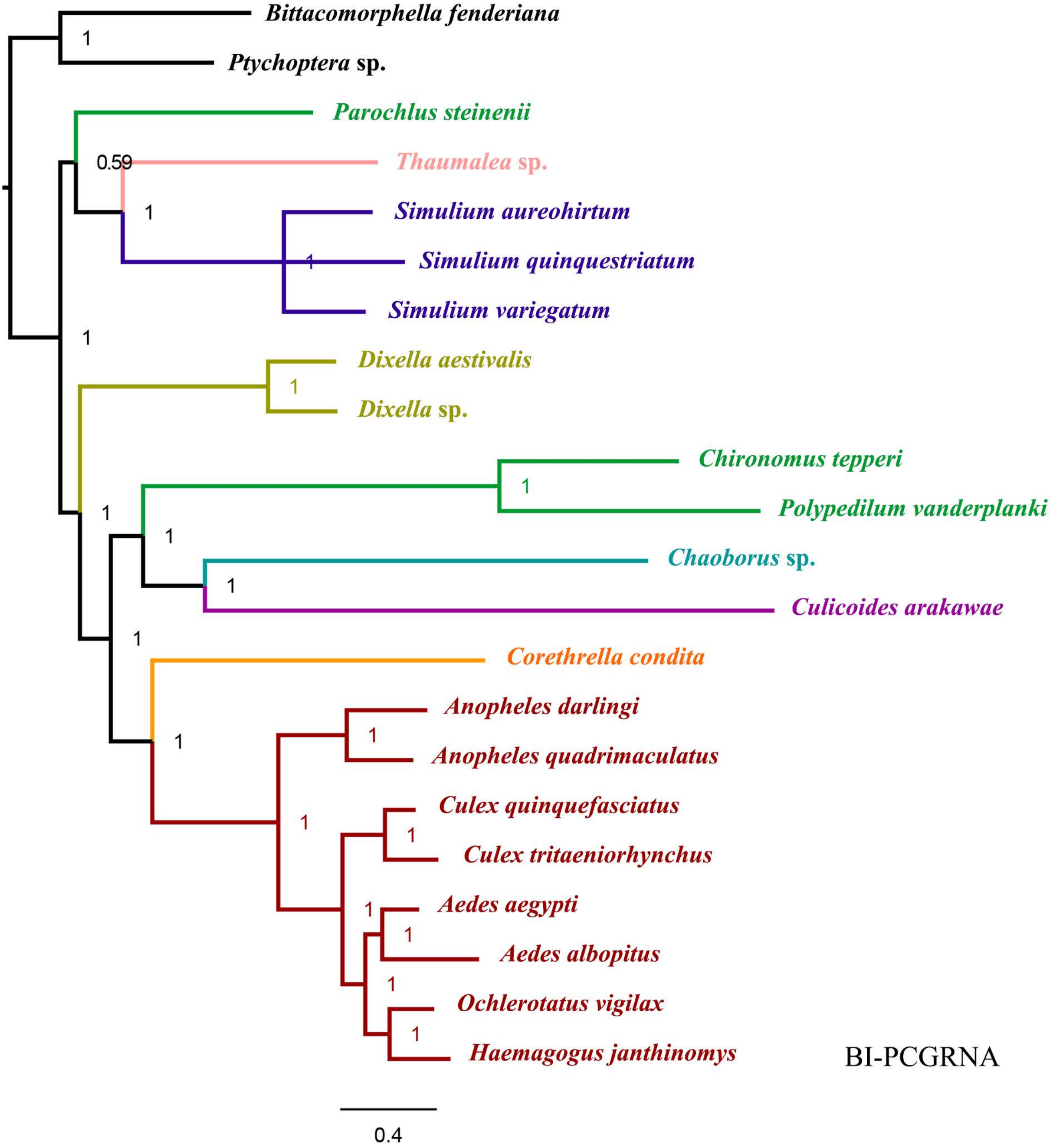

Bittacomorphella fenderiana
Ptychoptera sp.
Parochlus steinenii
Thaumalea sp.
Simulium aureohirtum
Simulium quinquestriatum
Simulium variegatum
Dixella aestivalis
Dixella sp.
Chironomus tepperi
Polypedilum vanderplanki
Chaoborus sp.
Culicoides arakawae
Corethrella condita
Anopheles darlingi
Anopheles quadrimaculatus
Culex quinquefasciatus
Culex tritaeniorhynchus
Aedes aegypti
Aedes albopitus
Ochlerotatus vigilax
Haemagogus janthinomys
BI-PCGRNA
0.59
0.4

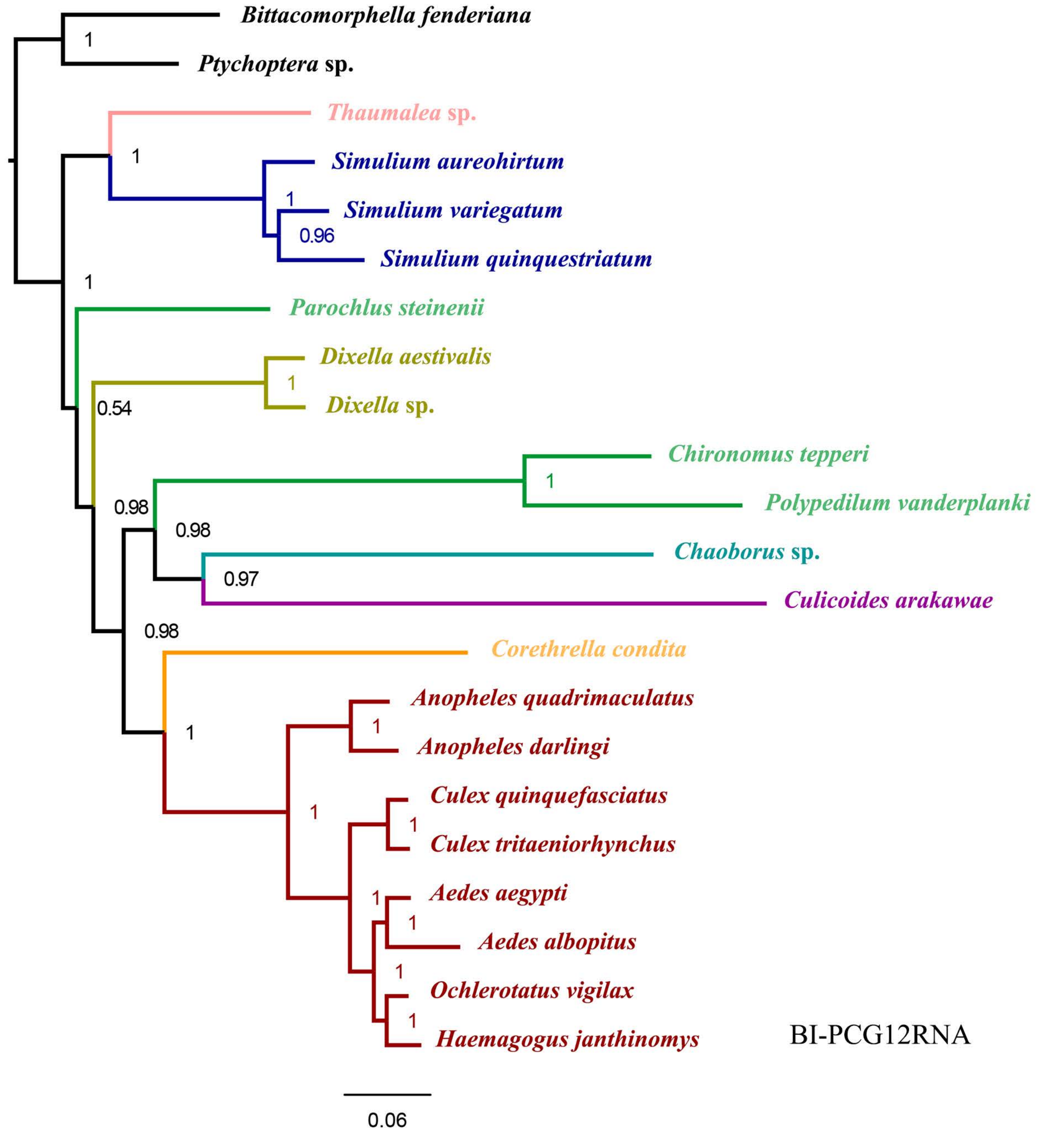
Bittacomorphella fenderiana
Ptychoptera sp.
Thaumalea sp.
Simulium aureohirtum
Simulium variegatum
Simulium quinquestriatum
Parochlus steinenii
Dixella aestivalis
Dixella sp.
Chironomus tepperi
Polypedilum vanderplanki
Chaoborus sp.
Culicoides arakawae
Corethrella condita
Anopheles quadrimaculatus
Anopheles darlingi
Culex quinquefasciatus
Culex tritaeniorhynchus
Aedes aegypti
Aedes albopitus
Ochlerotatus vigilax
Haemagogus janthinomys
BI-PCG12RNA
1
1
1
0.96
1
1
0.54
1
0.98
0.98
0.97
0.98
1
1
1
1
1
1
1
1
0.06

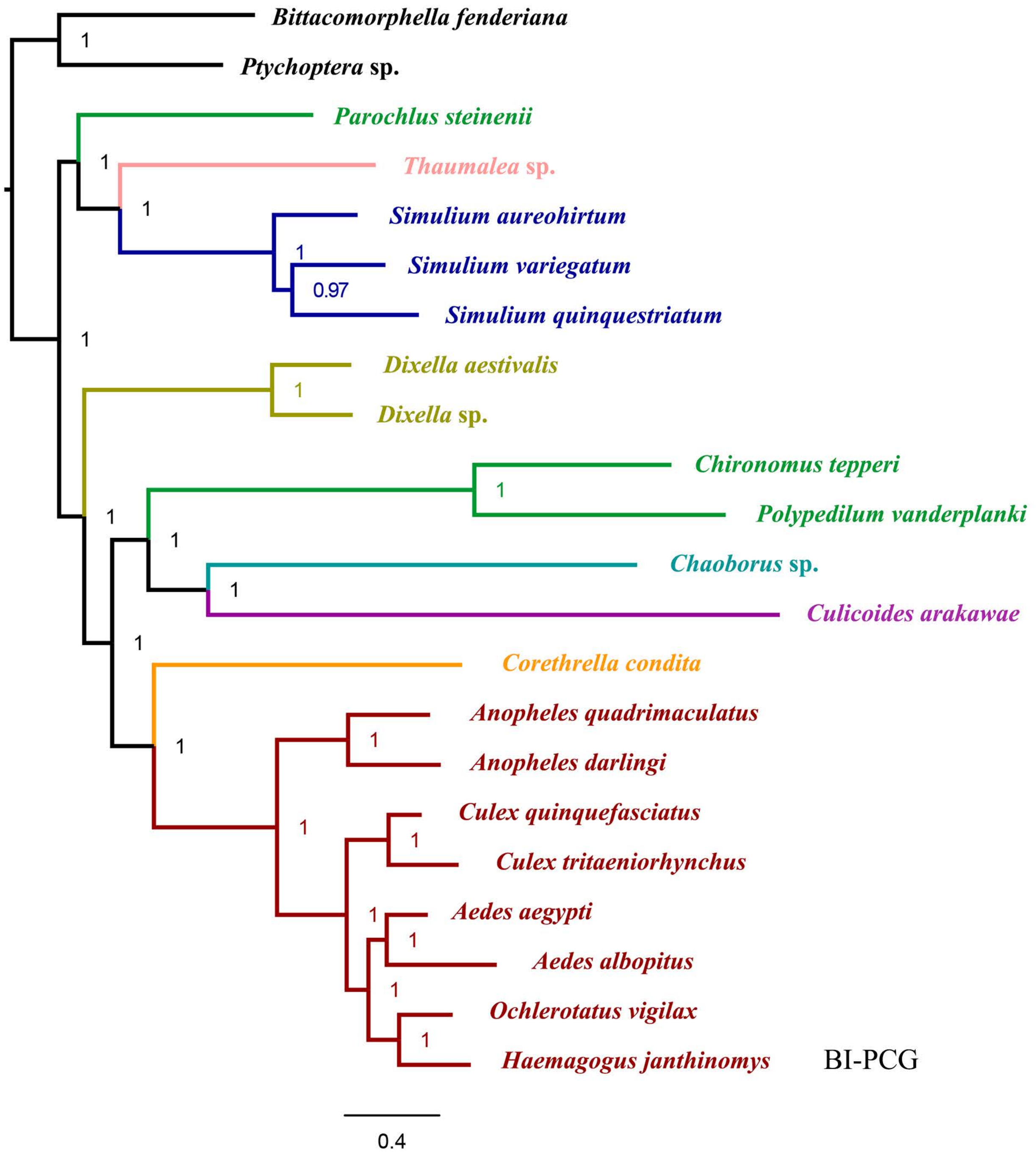
Bittacomorphella fenderiana
Ptychoptera sp.
Parochlus steinenii
Thaumalea sp.
Simulium aureohirtum
Simulium variegatum
Simulium quinquestriatum
Dixella aestivalis
Dixella sp.
Chironomus tepperi
Polypedilum vanderplanki
Chaoborus sp.
Culicoides arakawae
Corethrella condita
Anopheles quadrimaculatus
Anopheles darlingi
Culex quinquefasciatus
Culex tritaeniorhynchus
Aedes aegypti
Aedes albopitus
Ochlerotatus vigilax
Haemagogus janthinomys
BI-PCG
0.97
0.4

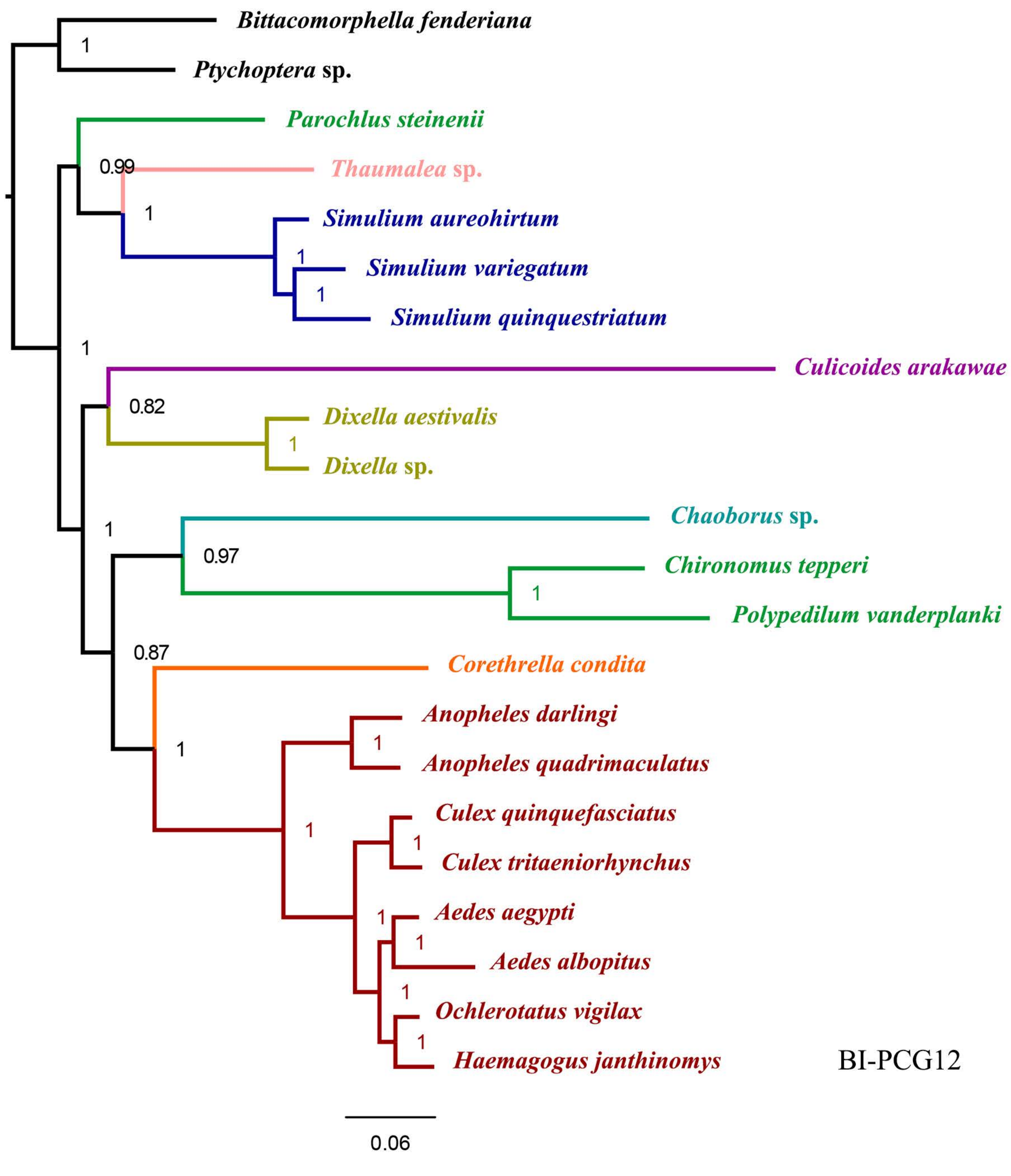

Bittacomorphella fenderiana
Ptychoptera sp.
Parochlus steinenii
Thaumalea sp.
Simulium aureohirtum
Simulium variegatum
Simulium quinquestriatum
Culicoides arakawae
Dixella aestivalis
Dixella sp.
Chaoborus sp.
Chironomus tepperi
Polypedilum vanderplanki
Corethrella condita
Anopheles darlingi
Anopheles quadrimaculatus
Culex quinquefasciatus
Culex tritaeniorhynchus
Aedes aegypti
Aedes albopitus
Ochlerotatus vigilax
Haemagogus janthinomys
1
0.99
1
1
1
1
0.82
1
1
0.97
1
0.87
1
1
1
1
1
1
1
1
BI-PCG12
0.06

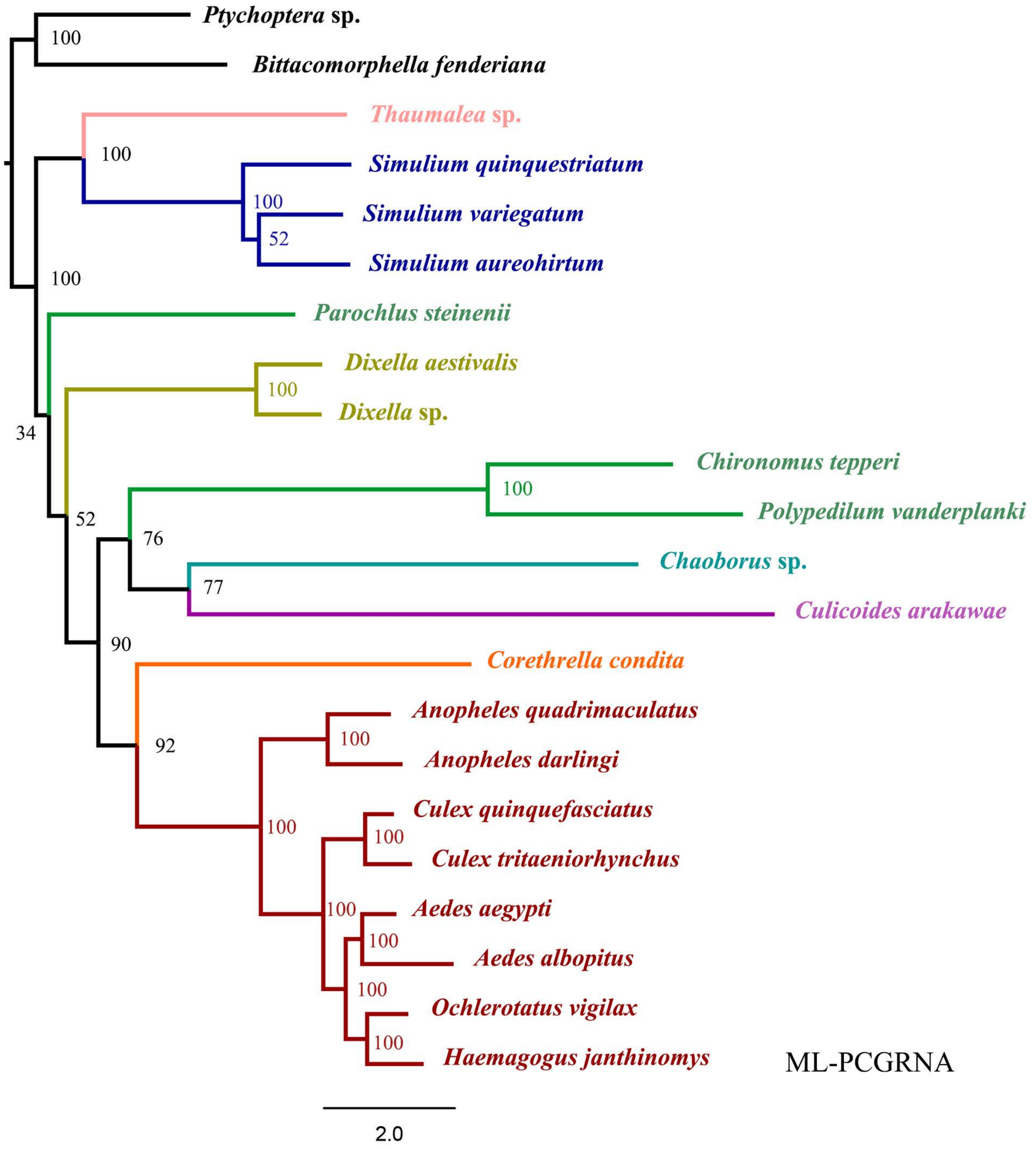
Ptychoptera sp.
Bittacomorphella fenderiana
Thaumalea sp.
Simulium quinquestriatum
Simulium variegatum
Simulium aureohirtum
Parochlus steinenii
Dixella aestivalis
Dixella sp.
Chironomus tepperi
Polypedilum vanderplanki
Chaoborus sp.
Culicoides arakawae
Corethrella condita
Anopheles quadrimaculatus
Anopheles darlingi
Culex quinquefasciatus
Culex tritaeniorhynchus
Aedes aegypti
Aedes albopitus
Ochlerotatus vigilax
Haemagogus janthinomys
100
100
100
52
100
100
34
52
100
76
77
90
92
100
100
100
100
100
100
100
ML-PCGRNA
2.0

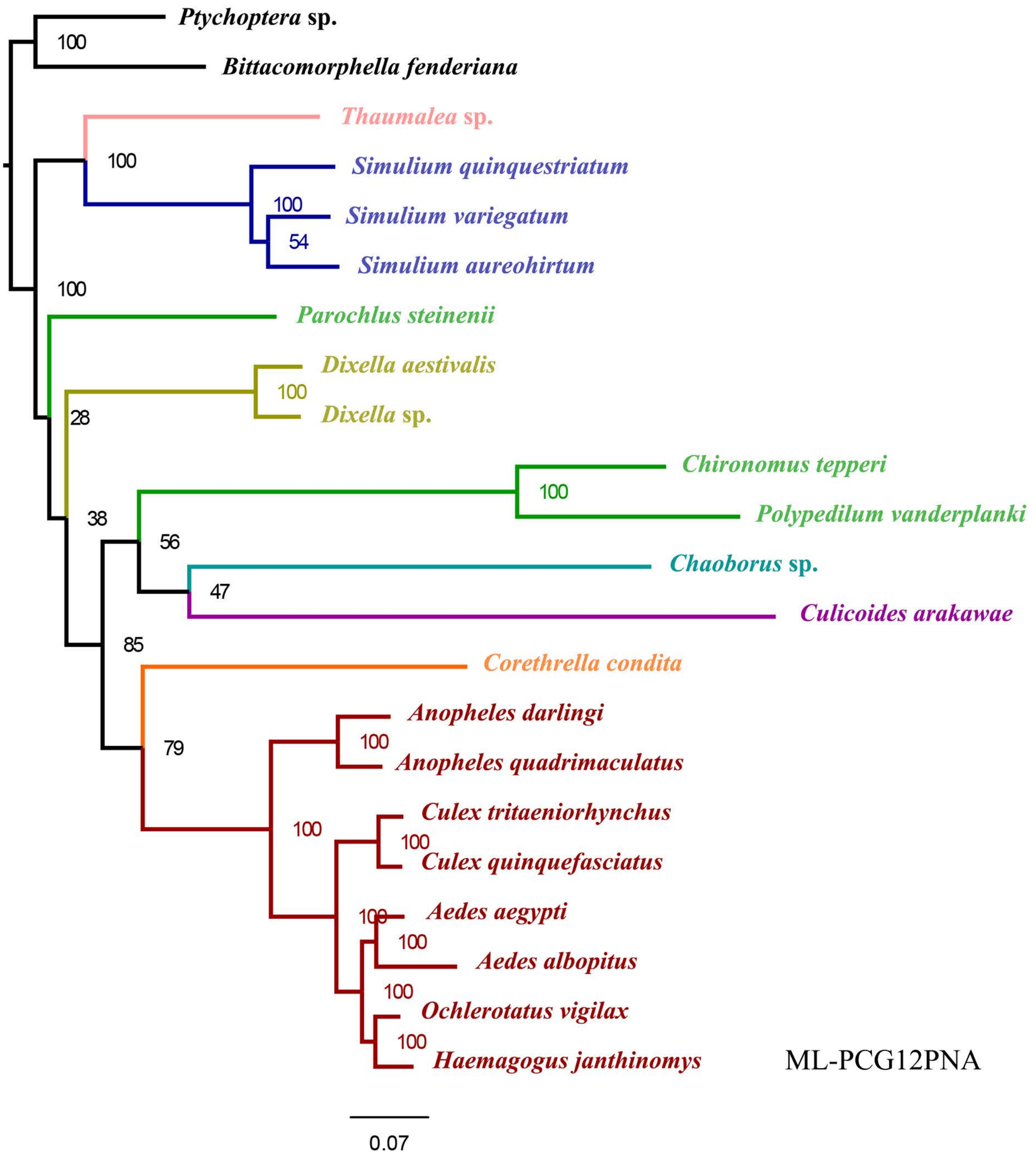
Ptychoptera sp.
Bittacomorphella fenderiana
Thaumalea sp.
Simulium quinquestriatum
Simulium variegatum
Simulium aureohirtum
Parochlus steinenii
Dixella aestivalis
Dixella sp.
Chironomus tepperi
Polypedilum vanderplanki
Chaoborus sp.
Culicoides arakawae
Corethrella condita
Anopheles darlingi
Anopheles quadrimaculatus
Culex tritaeniorhynchus
Culex quinquefasciatus
Aedes aegypti
Aedes albopitus
Ochlerotatus vigilax
Haemagogus janthinomys
ML-PCG12PNA
0.07

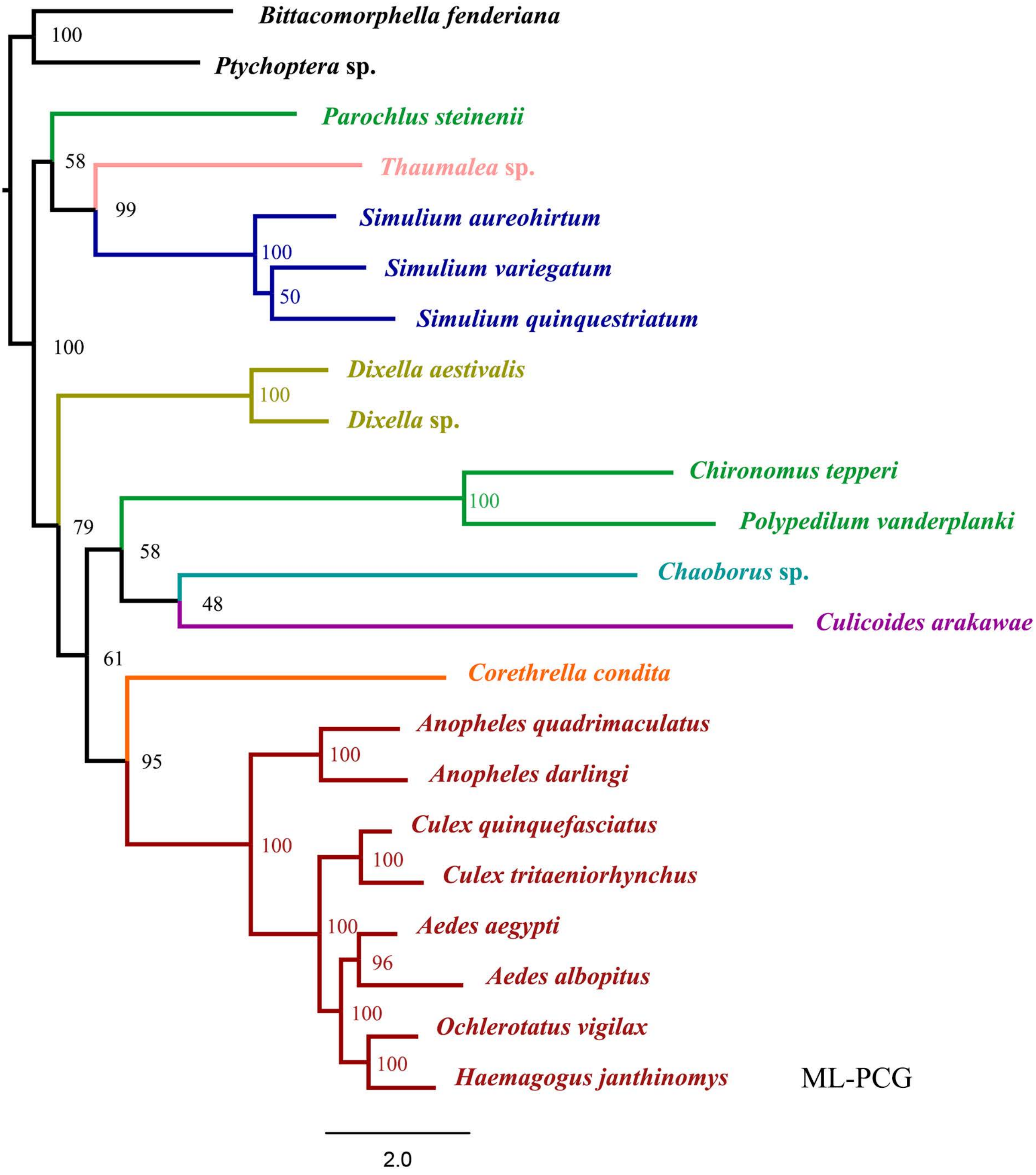
Bittacomorphella fenderiana
100
Ptychoptera sp.
Parochlus steinenii
58
Thaumalea sp.
99
Simulium aureohirtum
100
Simulium variegatum
50
Simulium quinquestriatum
100
Dixella aestivalis
100
Dixella sp.
Chironomus tepperi
100
Polypedilum vanderplanki
79
58
Chaoborus sp.
48
Culicoides arakawae
61
Corethrella condita
Anopheles quadrimaculatus
100
Anopheles darlingi
95
Culex quinquefasciatus
100
100
Culex tritaeniorhynchus
Aedes aegypti
100
96
Aedes albopitus
100
Ochlerotatus vigilax
100
Haemagogus janthinomys
ML-PCG
2.0

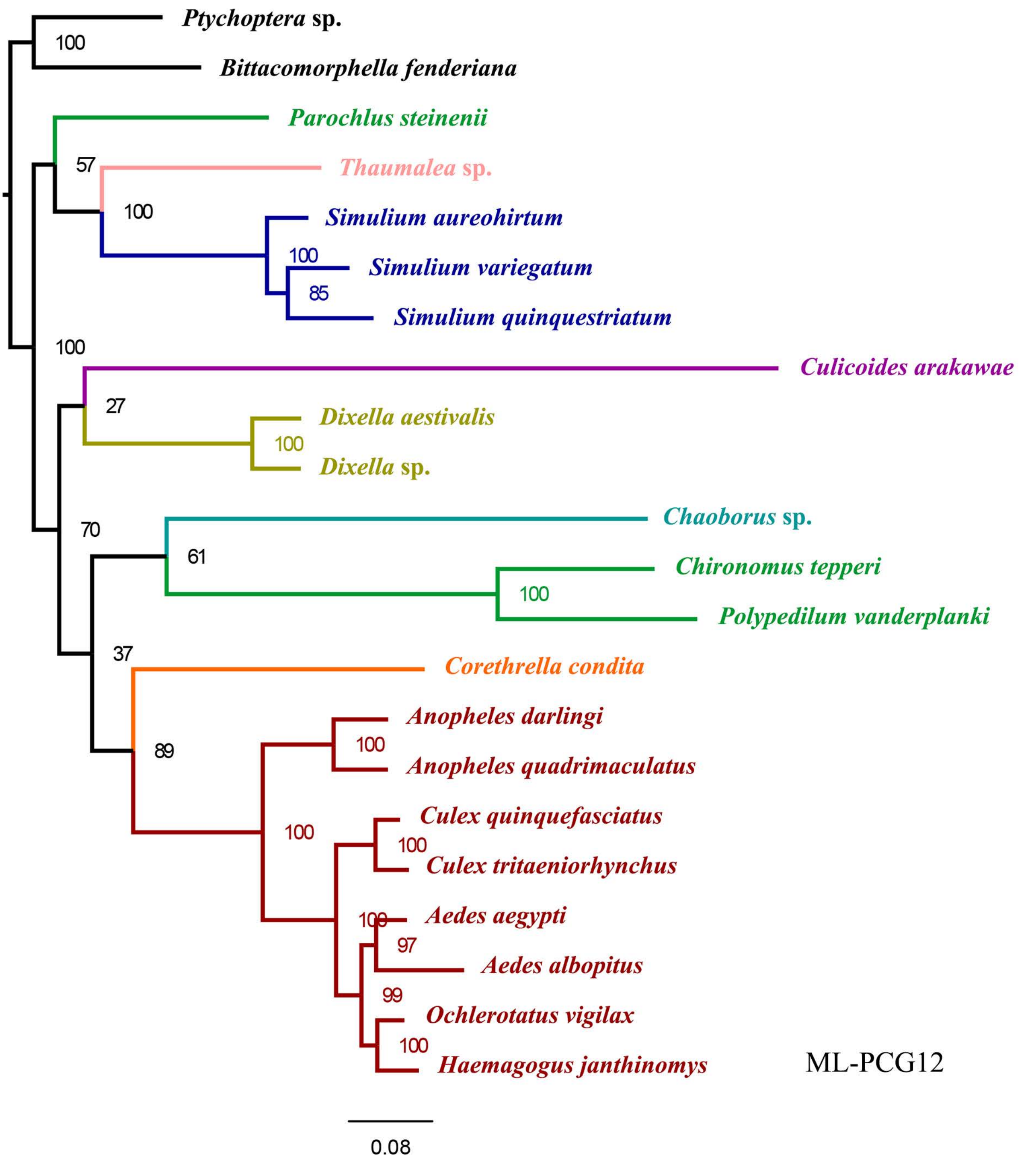
Ptychoptera sp.
100
Bittacomorphella fenderiana
Parochlus steinenii
57
Thaumalea sp.
100
Simulium aureohirtum
100
Simulium variegatum
85
Simulium quinquestriatum
100
Culicoides arakawae
27
Dixella aestivalis
100
Dixella sp.
Chaoborus sp.
70
61
Chironomus tepperi
100
Polypedilum vanderplanki
37
Corethrella condita
Anopheles darlingi
89
100
Anopheles quadrimaculatus
Culex quinquefasciatus
100
100
Culex tritaeniorhynchus
100
Aedes aegypti
97
Aedes albopitus
99
Ochlerotatus vigilax
100
Haemagogus janthinomys
ML-PCG12
0.08
