# Supplementary material for: Mitochondrial Genomes Provide Insights into the Phylogeny of Culicomorpha (Insecta: Diptera)
Source: Int J Mol Sci. 2019 Feb 11;20(3):747. doi: 10.3390/ijms20030747 (PMC6387087; doi:10.3390/ijms20030747)
Supplement: Supplementary file 1 [file ijms-20-00747-s001.zip › Supplementary Files/Figure S2.pdf]

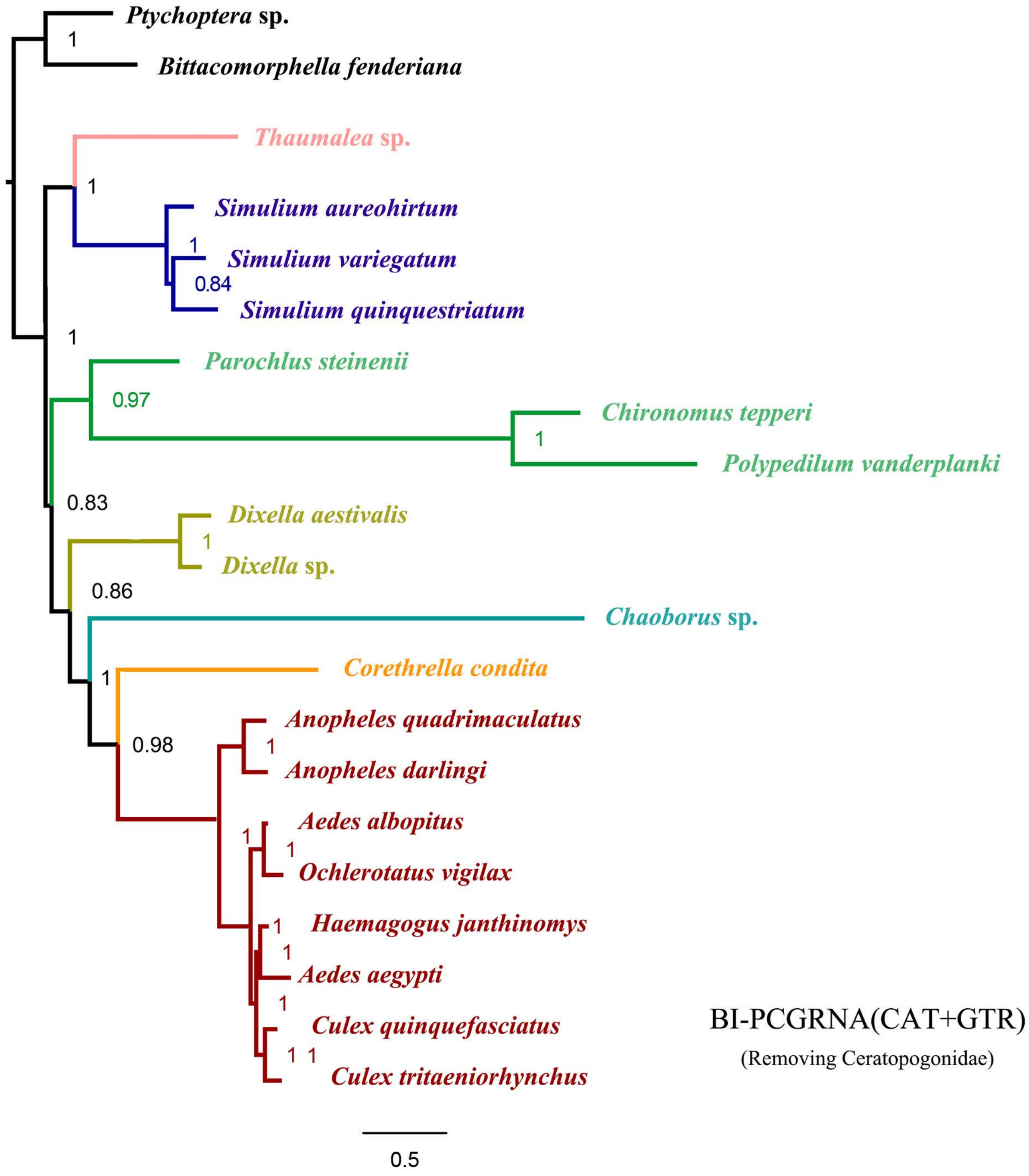

Ptychoptera sp.
1
Bittacomorphella fenderiana
Thaumalea sp.
1
Simulium aureohirtum
1
Simulium variegatum
0.84
Simulium quinquestriatum
1
Parochlus steinenii
0.97
Chironomus tepperi
1
Polypedilum vanderplanki
0.83
Dixella aestivalis
1
Dixella sp.
0.86
Chaoborus sp.
1
Corethrella condita
0.98
Anopheles quadrimaculatus
1
Anopheles darlingi
1
Aedes albopitus
1
Ochlerotatus vigilax
1
Haemagogus janthinomys
1
Aedes aegypti
1
Culex quinquefasciatus
1 1
Culex tritaeniorhynchus
BI-PCGRNA(CAT+GTR)
(Removing Ceratopogonidae)
0.5

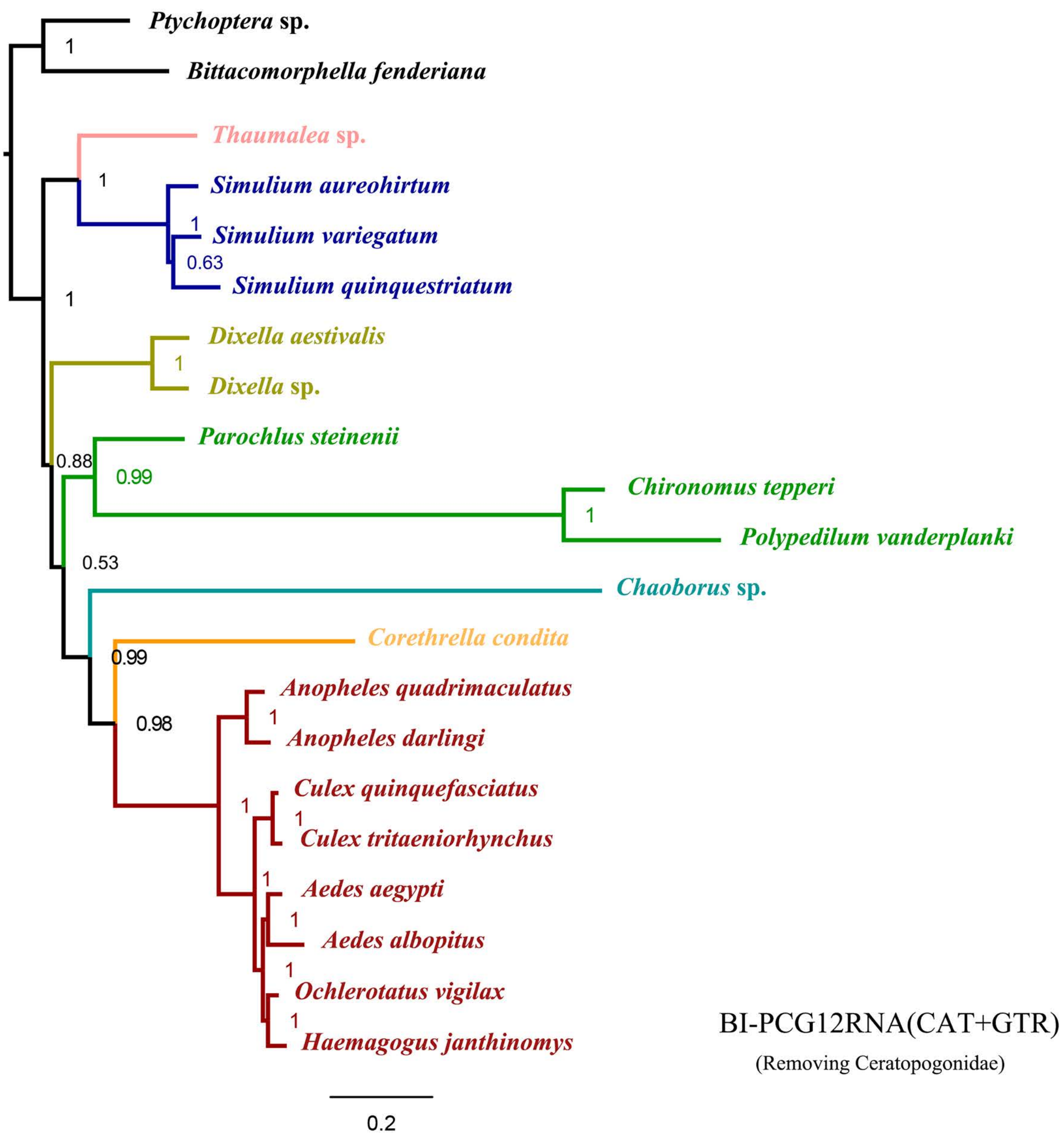

Ptychoptera sp.
Bittacomorphella fenderiana
Thaumalea sp.
Simulium aureohirtum
Simulium variegatum
Simulium quinquestriatum
Dixella aestivalis
Dixella sp.
Parochlus steinenii
Chironomus tepperi
Polypedilum vanderplanki
Chaoborus sp.
Corethrella condita
Anopheles quadrimaculatus
Anopheles darlingi
Culex quinquefasciatus
Culex tritaeniorhynchus
Aedes aegypti
Aedes albopitus
Ochlerotatus vigilax
Haemagogus janthinomys
1
1
1
0.63
1
1
0.88
0.99
1
0.53
0.99
0.98
1
1
1
1
1
1
1
0.2
BI-PCG12RNA(CAT+GTR)
(Removing Ceratopogonidae)

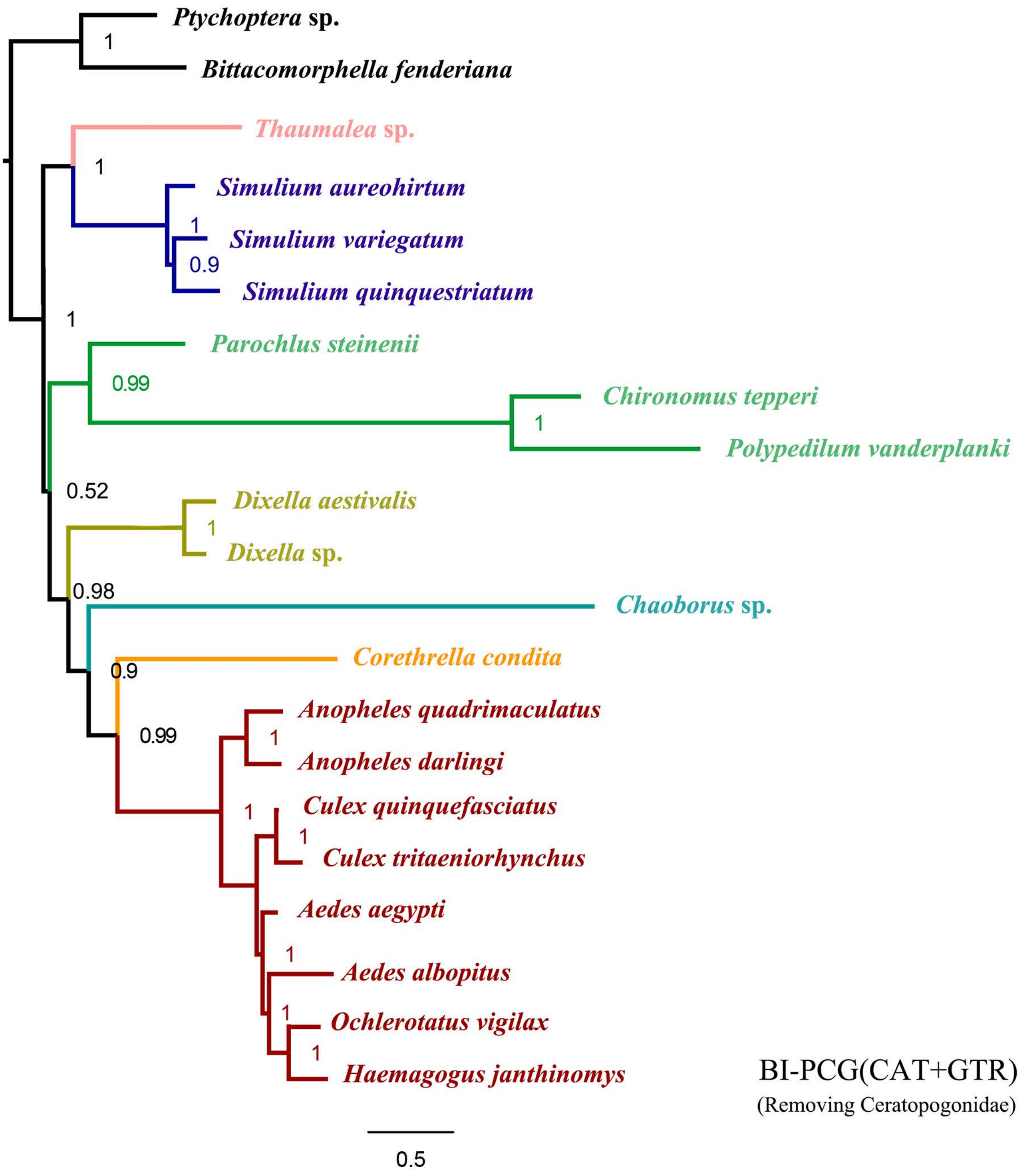

Ptychoptera sp.
Bittacomorphella fenderiana
Thaumalea sp.
Simulium aureohirtum
Simulium variegatum
Simulium quinquestriatum
Parochlus steinenii
Chironomus tepperi
Polypedilum vanderplanki
Dixella aestivalis
Dixella sp.
Chaoborus sp.
Corethrella condita
Anopheles quadrimaculatus
Anopheles darlingi
Culex quinquefasciatus
Culex tritaeniorhynchus
Aedes aegypti
Aedes albopitus
Ochlerotatus vigilax
Haemagogus janthinomys
1
1
1
0.9
1
0.99
1
0.52
1
0.98
0.9
0.99
1
1
1
1
1
1
0.5
BI-PCG(CAT+GTR)
(Removing Ceratopogonidae)

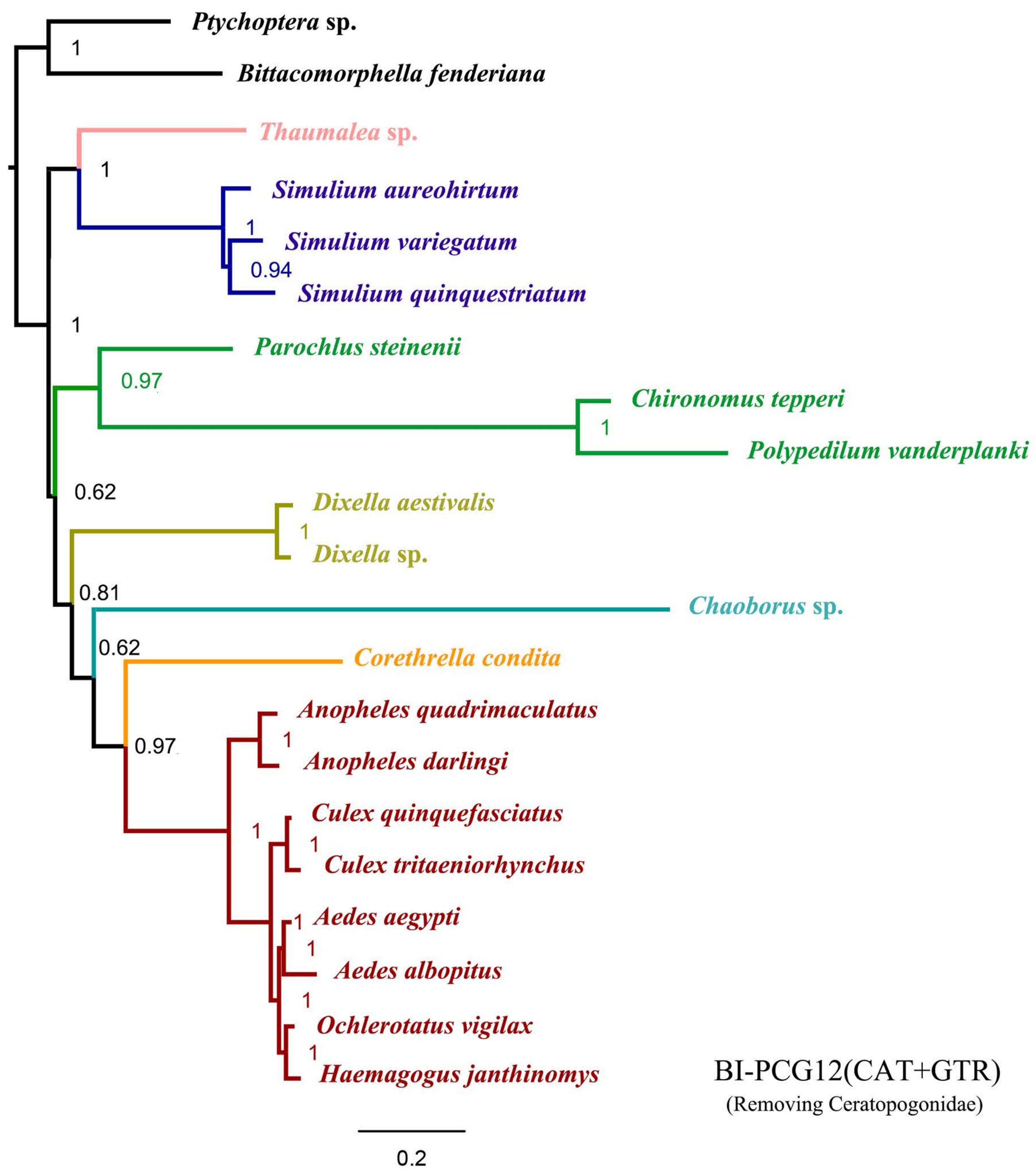

Ptychoptera sp.
Bittacomorphella fenderiana
Thaumalea sp.
Simulium aureohirtum
Simulium variegatum
Simulium quinquestriatum
Parochlus steinenii
Chironomus tepperi
Polypedilum vanderplanki
Dixella aestivalis
Dixella sp.
Chaoborus sp.
Corethrella condita
Anopheles quadrimaculatus
Anopheles darlingi
Culex quinquefasciatus
Culex tritaeniorhynchus
Aedes aegypti
Aedes albopitus
Ochlerotatus vigilax
Haemagogus janthinomys
1
1
1
0.94
1
0.97
1
0.62
1
0.81
0.62
0.97
1
1
1
1
1
1
1
0.2
BI-PCG12(CAT+GTR)
(Removing Ceratopogonidae)

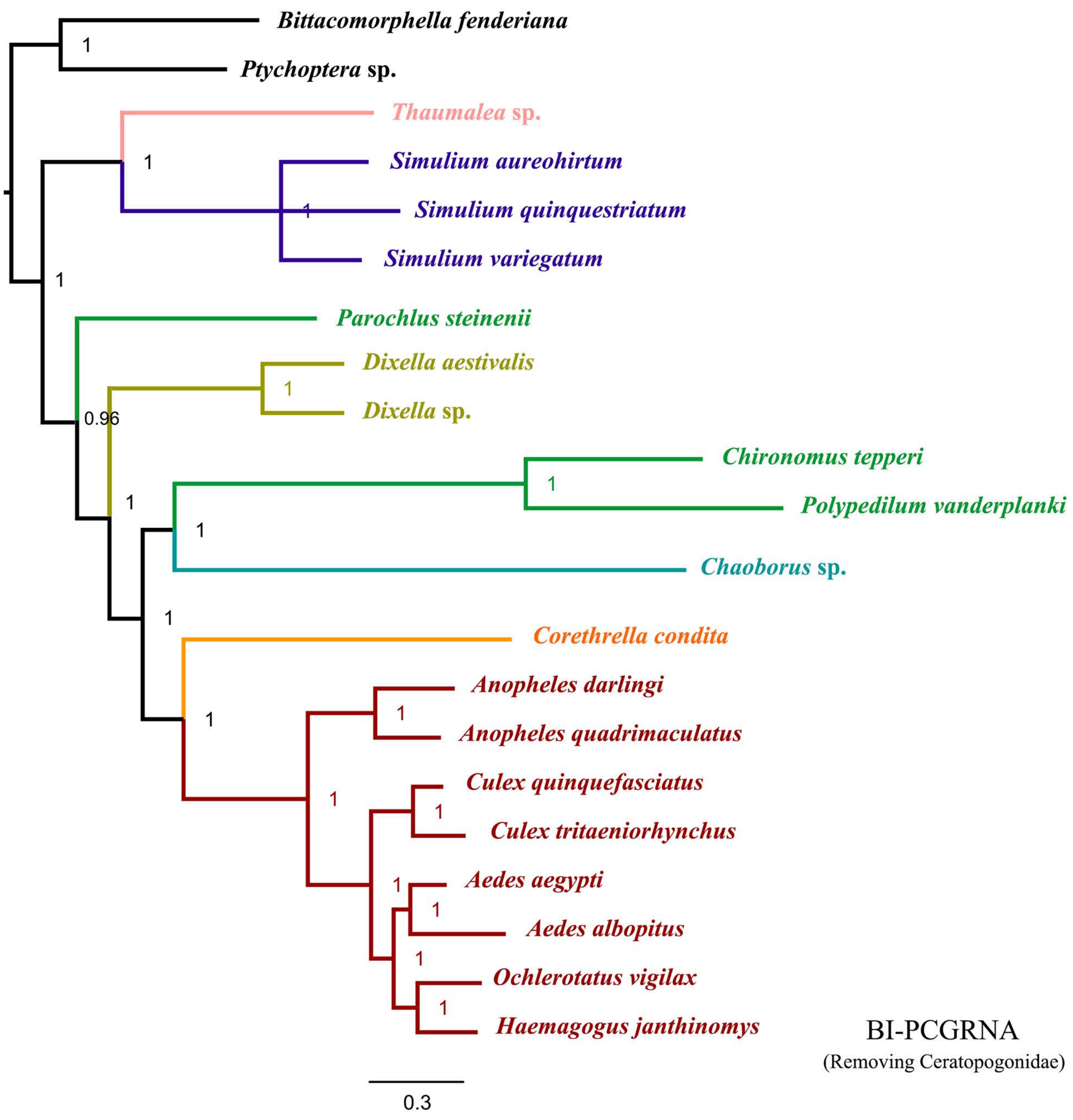

Bittacomorphella fenderiana
Ptychoptera sp.
Thaumalea sp.
Simulium aureohirtum
Simulium quinquestriatum
Simulium variegatum
Parochlus steinenii
Dixella aestivalis
Dixella sp.
Chironomus tepperi
Polypedilum vanderplanki
Chaoborus sp.
Corethrella condita
Anopheles darlingi
Anopheles quadrimaculatus
Culex quinquefasciatus
Culex tritaeniorhynchus
Aedes aegypti
Aedes albopitus
Ochlerotatus vigilax
Haemagogus janthinomys
0.96
0.3
BI-PCGRNA
(Removing Ceratopogonidae)

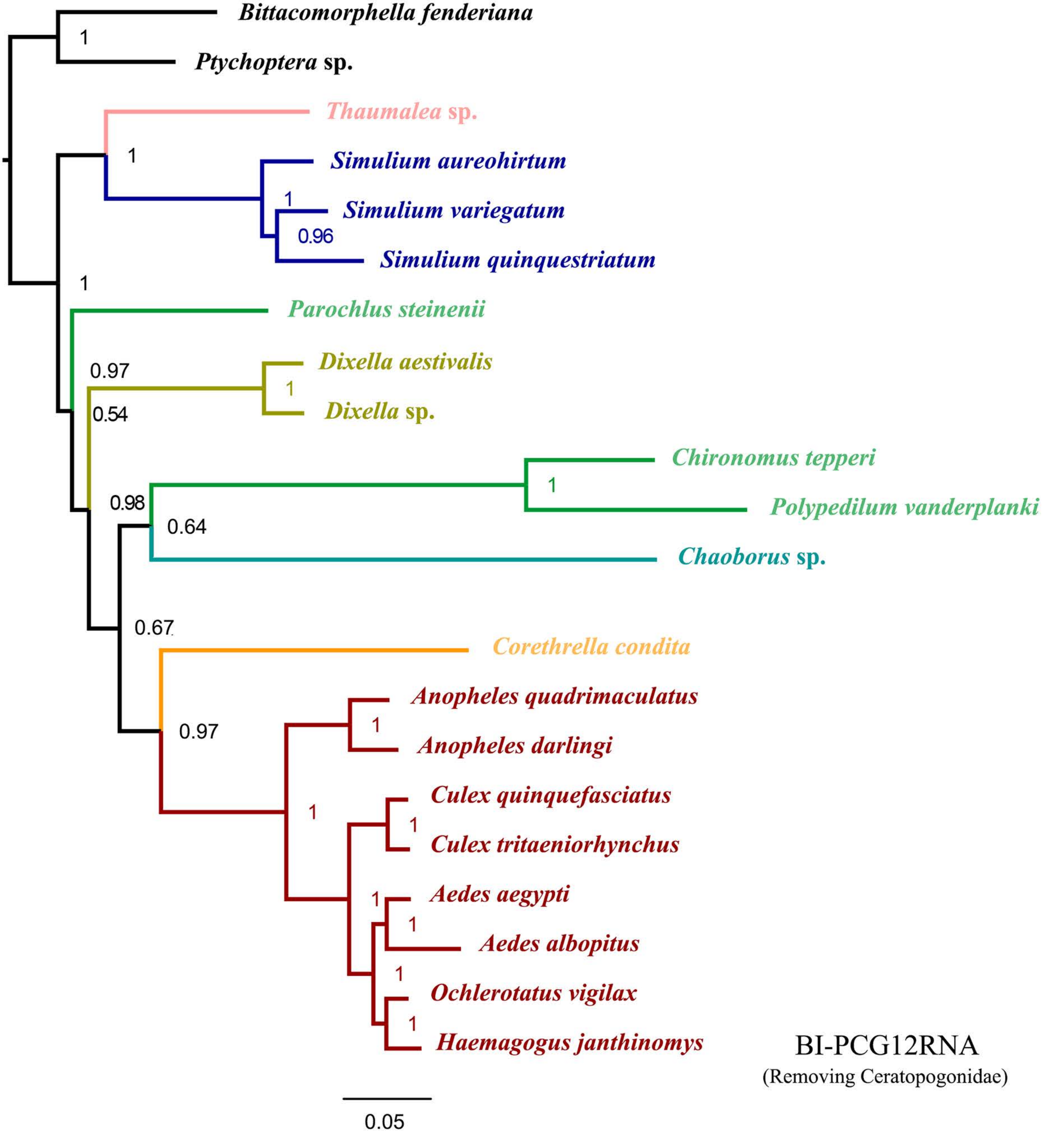

Bittacomorphella fenderiana
Ptychoptera sp.
Thaumalea sp.
Simulium aureohirtum
Simulium variegatum
Simulium quinquestriatum
Parochlus steinenii
Dixella aestivalis
Dixella sp.
Chironomus tepperi
Polypedilum vanderplanki
Chaoborus sp.
Corethrella condita
Anopheles quadrimaculatus
Anopheles darlingi
Culex quinquefasciatus
Culex tritaeniorhynchus
Aedes aegypti
Aedes albopitus
Ochlerotatus vigilax
Haemagogus janthinomys
1
1
1
0.96
1
0.97
1
0.54
0.98
1
0.64
0.67
0.97
1
1
1
1
1
1
1
BI-PCG12RNA
(Removing Ceratopogonidae)
0.05

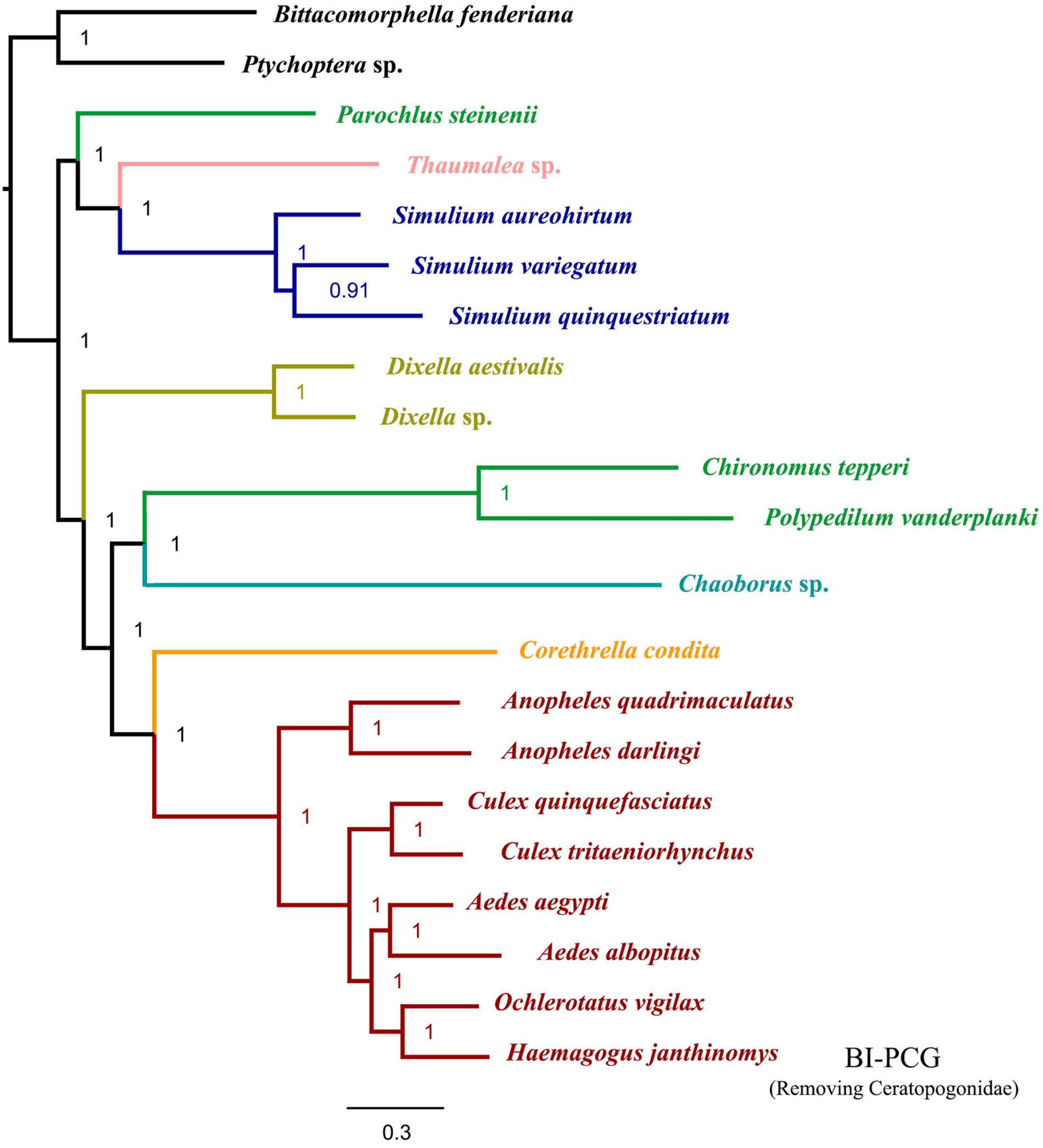
Bittacomorphella fenderiana
Ptychoptera sp.
Parochlus steinenii
Thaumalea sp.
Simulium aureohirtum
Simulium variegatum
Simulium quinquestriatum
Dixella aestivalis
Dixella sp.
Chironomus tepperi
Polypedilum vanderplanki
Chaoborus sp.
Corethrella condita
Anopheles quadrimaculatus
Anopheles darlingi
Culex quinquefasciatus
Culex tritaeniorhynchus
Aedes aegypti
Aedes albopitus
Ochlerotatus vigilax
Haemagogus janthinomys
1
0.91
0.3
BI-PCG
(Removing Ceratopogonidae)

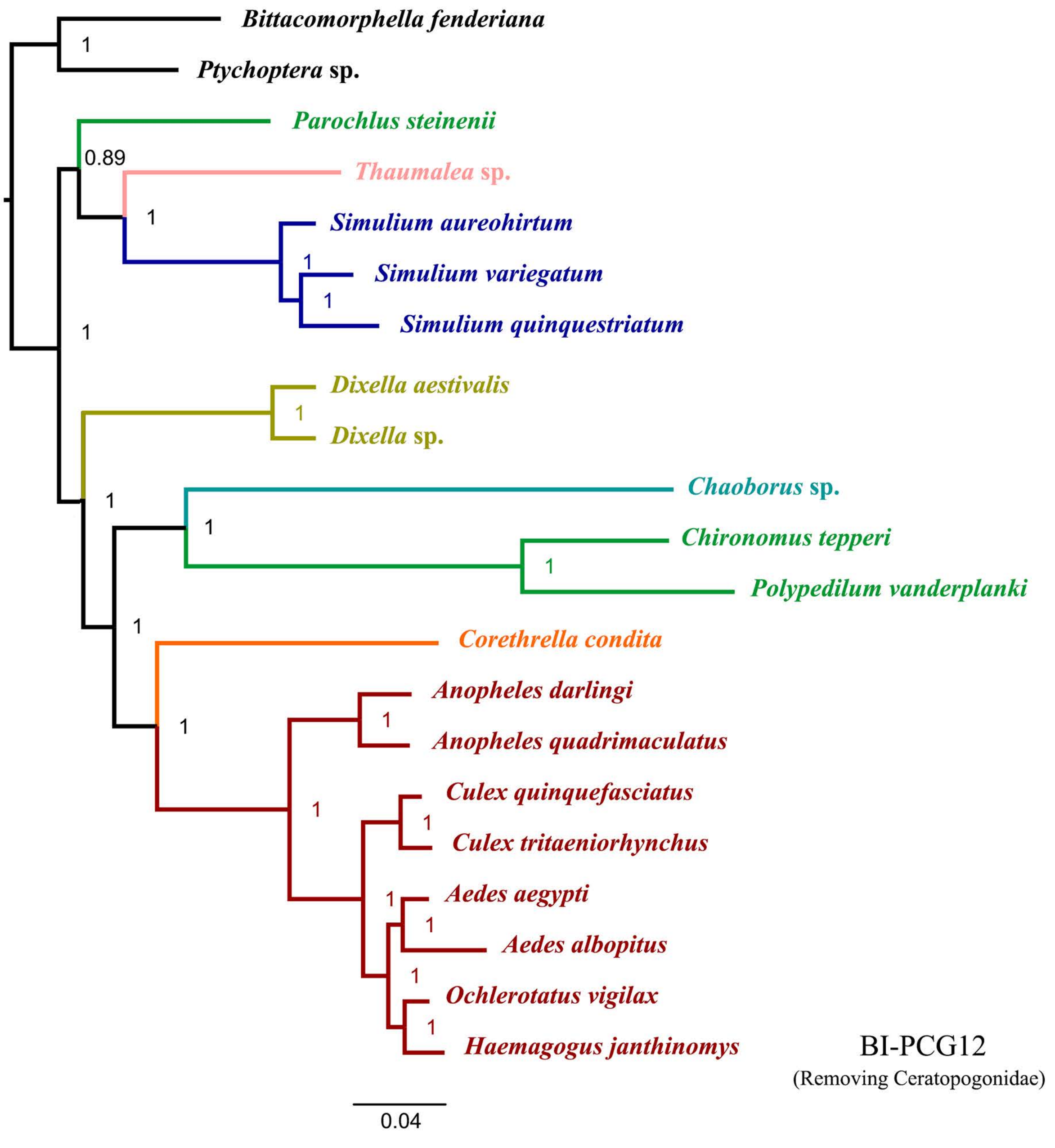
Bittacomorphella fenderiana
Ptychoptera sp.
Parochlus steinenii
Thaumalea sp.
Simulium aureohirtum
Simulium variegatum
Simulium quinquestriatum
Dixella aestivalis
Dixella sp.
Chaoborus sp.
Chironomus tepperi
Polypedilum vanderplanki
Corethrella condita
Anopheles darlingi
Anopheles quadrimaculatus
Culex quinquefasciatus
Culex tritaeniorhynchus
Aedes aegypti
Aedes albopitus
Ochlerotatus vigilax
Haemagogus janthinomys
1
0.89
1
1
1
1
1
1
1
1
1
1
1
1
1
1
1
1
1
0.04
BI-PCG12
(Removing Ceratopogonidae)

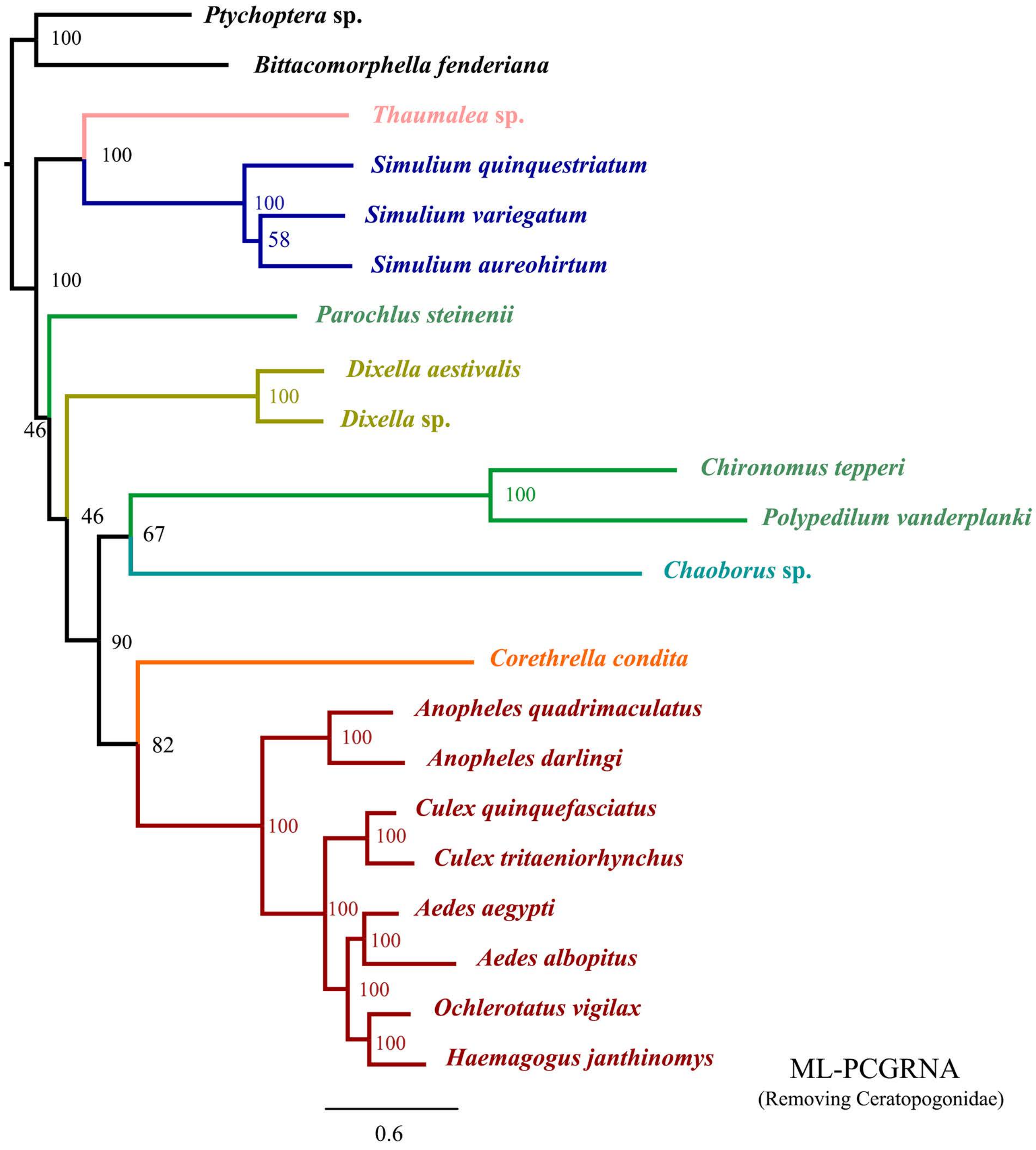

Ptychoptera sp.
100
Bittacomorphella fenderiana
Thaumalea sp.
100
Simulium quinquestriatum
100
Simulium variegatum
58
Simulium aureohirtum
100
Parochlus steinenii
Dixella aestivalis
100
Dixella sp.
46
Chironomus tepperi
100
Polypedilum vanderplanki
46
67
Chaoborus sp.
90
Corethrella condita
82
Anopheles quadrimaculatus
100
Anopheles darlingi
Culex quinquefasciatus
100
100
Culex tritaeniorhynchus
Aedes aegypti
100
100
Aedes albopitus
100
Ochlerotatus vigilax
100
Haemagogus janthinomys
ML-PCGRNA
(Removing Ceratopogonidae)
0.6

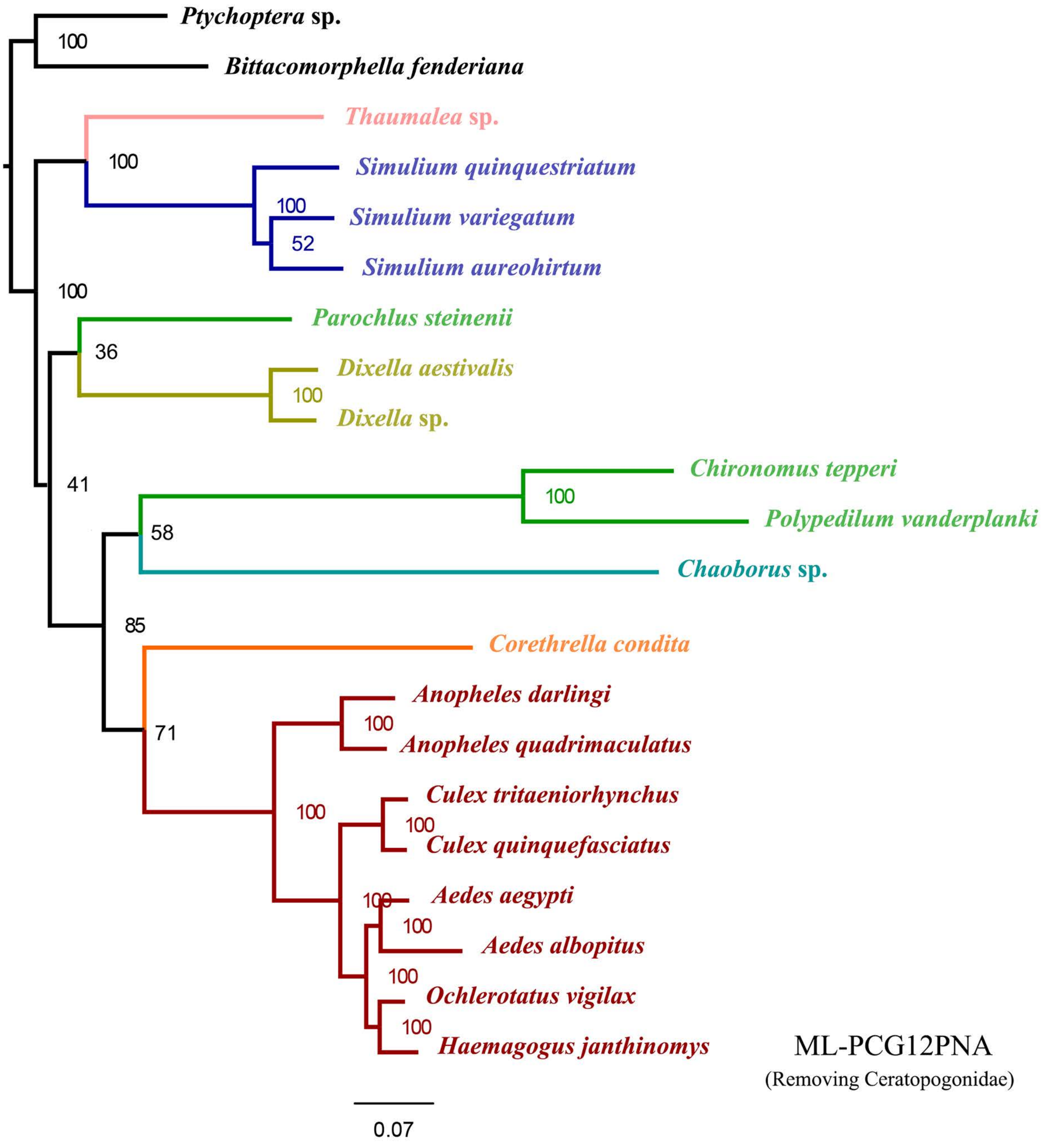
Ptychoptera sp.
Bittacomorphella fenderiana
Thaumalea sp.
Simulium quinquestriatum
Simulium variegatum
Simulium aureohirtum
Parochlus steinenii
Dixella aestivalis
Dixella sp.
Chironomus tepperi
Polypedilum vanderplanki
Chaoborus sp.
Corethrella condita
Anopheles darlingi
Anopheles quadrimaculatus
Culex tritaeniorhynchus
Culex quinquefasciatus
Aedes aegypti
Aedes albopitus
Ochlerotatus vigilax
Haemagogus janthinomys
100
100
100
52
100
36
100
41
100
58
85
71
100
100
100
100
100
100
100
0.07
ML-PCG12PNA
(Removing Ceratopogonidae)

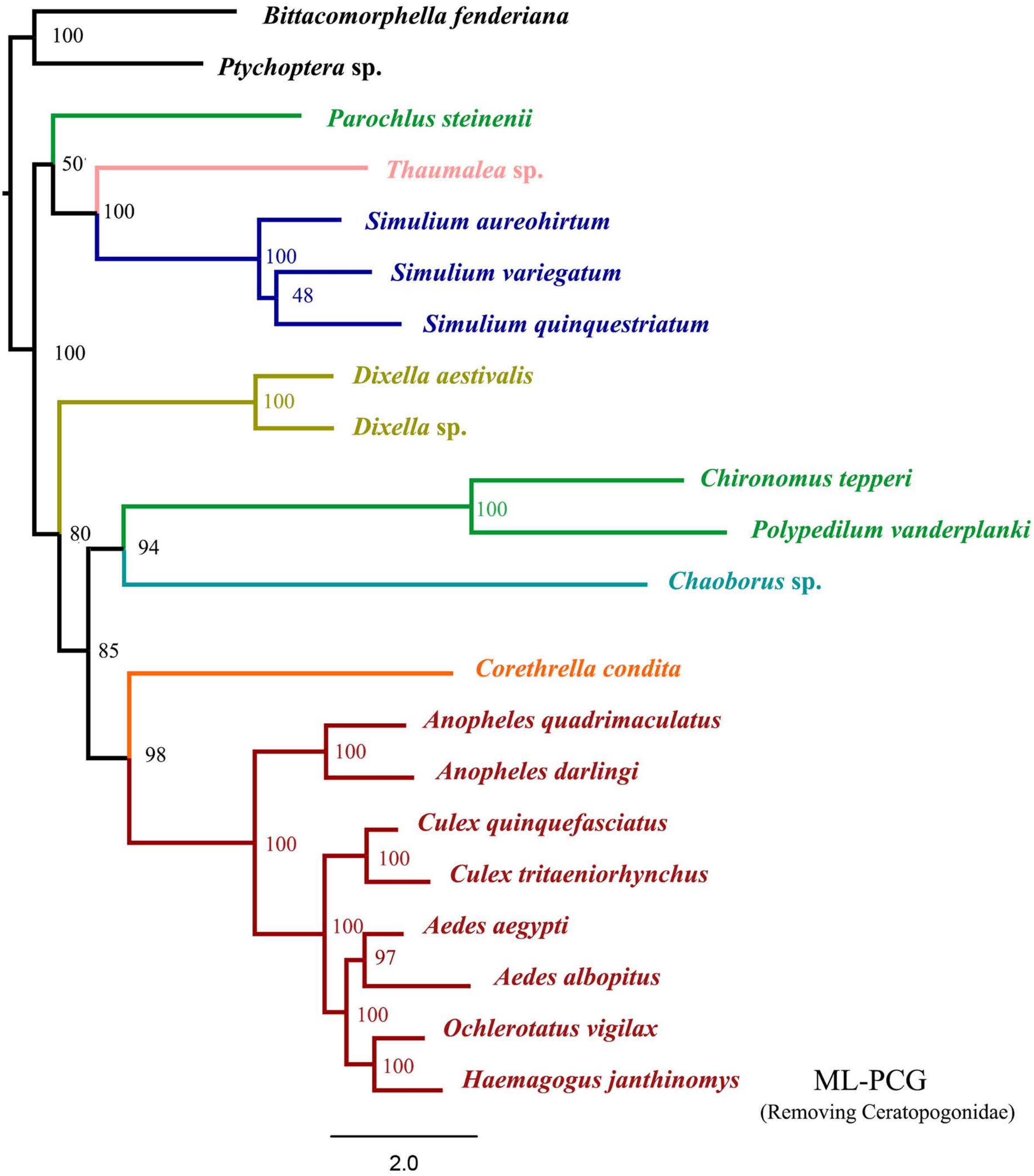

Bittacomorphella fenderiana
Ptychoptera sp.
Parochlus steinenii
Thaumalea sp.
Simulium aureohirtum
Simulium variegatum
Simulium quinquestriatum
Dixella aestivalis
Dixella sp.
Chironomus tepperi
Polypedilum vanderplanki
Chaoborus sp.
Corethrella condita
Anopheles quadrimaculatus
Anopheles darlingi
Culex quinquefasciatus
Culex tritaeniorhynchus
Aedes aegypti
Aedes albopitus
Ochlerotatus vigilax
Haemagogus janthinomys
100
50
100
100
48
100
100
100
80
94
85
98
100
100
100
100
97
100
100
ML-PCG
(Removing Ceratopogonidae)
2.0

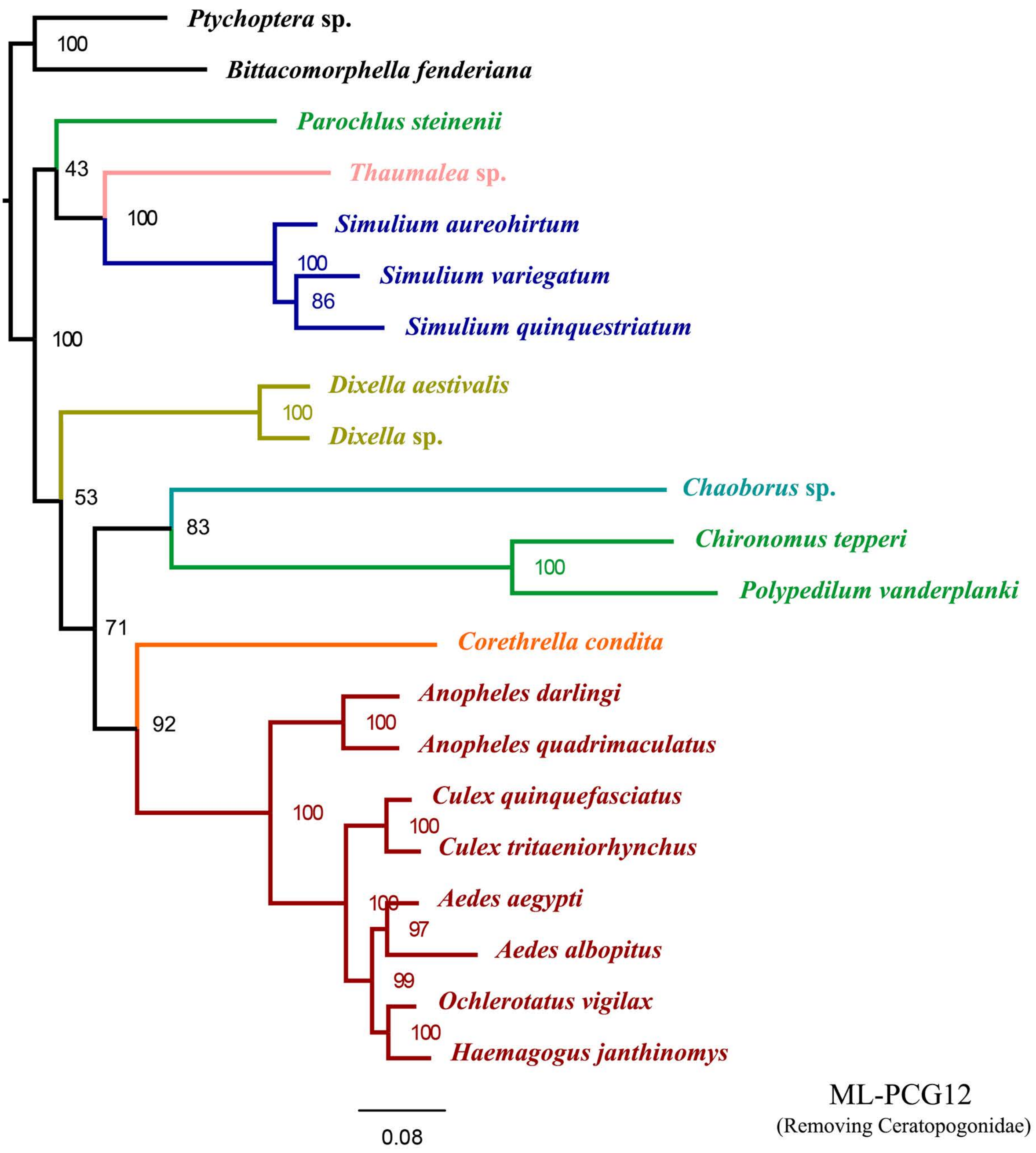

Ptychoptera sp.
100
Bittacomorphella fenderiana
Parochlus steinenii
43
Thaumalea sp.
100
Simulium aureohirtum
100
Simulium variegatum
86
Simulium quinquestriatum
100
Dixella aestivalis
100
Dixella sp.
53
Chaoborus sp.
83
Chironomus tepperi
100
Polypedilum vanderplanki
71
Corethrella condita
92
Anopheles darlingi
100
Anopheles quadrimaculatus
Culex quinquefasciatus
100
100
Culex tritaeniorhynchus
Aedes aegypti
100
97
Aedes albopitus
99
Ochlerotatus vigilax
100
Haemagogus janthinomys
0.08
ML-PCG12
(Removing Ceratopogonidae)
