# Supplementary material for: Mitochondrial Genomes Provide Insights into the Phylogeny of Culicomorpha (Insecta: Diptera)
Source: Int J Mol Sci. 2019 Feb 11;20(3):747. doi: 10.3390/ijms20030747 (PMC6387087; doi:10.3390/ijms20030747)
Supplement: Supplementary file 1 [file ijms-20-00747-s001.zip › Supplementary Files/Table S1-S6 .docx]

Table S1. Collection information of specimens.

| Infraorder | Family | Species | Collection data |
| --- | --- | --- | --- |
| Culicomorpha | Chaoboridae | *Chaoborus* sp.  ( pupa) | Kashii, Fukuoka-shi, Fukuoka Pref., Kyushu, Japan. October 2, 2013, Shigetaka Nonaka |
|  | Corethrellidae | *Corethrella condita* | MS, Washington Co. Greenville Cypress Preserve, 2015.IX.16 (CDC trap), G.R. Curler |
|  | Thaumaleidae | *Thaumalea* sp. | China, shaanxi, Zhashui, Shagou, 2014.VII.28, Chufei Tang |
|  | Simuliidae | *Simulium quinquestriatum* | China, Yunnan, Lvchun, Huanglian Mountain(1302M), 2013.VII.17，Feiyang Liang |

Table S2. Primer sequences used to amplify mitochondrial genomes of Culicomorpha

| NO. | Primer Name | Primer sequence (5’-3’) | Size（bp） |
| --- | --- | --- | --- |
| T01 | TM-J-206 | GCTAAATAAGCTAACAGGTTCAT | 550 |
|  | N2-N-732 | AAGGAAGTTTGGTTTAAACCTCC |  |
| T02 | N2-J-283 | CATGATTAGGAGCTTGAATAGG | 1200 |
|  | C1-N-1740 | AAAGATAGAGCAGGAGGTAA |  |
| 20 | SPB-586 | CCATTCCATTTYTGATTTCC | 1200 |
|  | SPB-1738 | TTTATTCGTGGAAATGCTATGTC |  |
| T03 | TY-J-1460 | TACAATCTATCGCCTAAACTTCAGCC | 700 |
|  | C1-N-2191 | CCCGGTAAAATTAAAATATAAACTTC |  |
| T04 | C1-J-1751 | GGAGCTCCTGATATAGCATTCCC | 1300 |
|  | TL2-N-3014 | TCCATTGCACTAATCTGCCATATTA |  |
| T05 | Cl-J-2183 | CAACATTTATTTTGATTTTTTGG | 3200 |
|  | C3-N-5460 | TCAACAAAGTGTCAGTATCATGC |  |
| 22 | SPB-2756 | ACATTTTTTCCTCAACATTT | 700 |
|  | SPB-3389 | TATTCATATCTTCAATATCATTGATG |  |
| T06 | C2-J-3530 | AAGTTGATGGAACTCCTGGACG | 1000 |
|  | A6-N-4493 | CTGTTAATCGAACTGCTAAAGTTC |  |
| T07 | C3-J-5005 | CTCCAGCAATTGAATTAGGAGCTA | 1100 |
|  | E-rev | AGTGATAAGCCTCTTTTTGGCTTC |  |
| T08 | F-fw | CATTTGATTTGCATTCAAAAAGTATTG | 1700 |
|  | N5-N-7707 | AGGATGAGATGGATTAGGACTAG |  |
| T09 | H-fw | GAAACAGGAGTAGGAGCTGC | 1200 |
|  | N4-N-8718 | GCTTATTCATCTGTTGCTCA |  |
| 09 | SPA-7572 | AAACGGAAACTGAGCTCTCTTAGT | 1300 |
|  | SPA-8727 | AAATCTTTAATTGCCTATTCTTC |  |
| T10 | I-fw | CTATTTAATAAAGAAATTTCTCC | 450 |
|  | N4-N-8924 | CCTAAAGCTCATGTTGAAGCTCC |  |
| T11 | N4-J-8614 | TGAGCAACAGAAGAATAAGC | 400 |
|  | N4-N-9061 | ATCAACCTGAACGATTACAAG |  |
| T12 | N4-J-8944 | CAGGAGCTTCAACATGAGCTTTAGG | 1000 |
|  | I-rev | CTTATTTTTGATTTACAAGACCAATG |  |
| T13 | N4-J-9511 | CCAAAATTGATAACCCTAAAGC | 1700 |
|  | CB-N-11218 | TCAGGTTGAATGTGAATTGG |  |
| T14 | CB-J-10933 | TATGTTCTACCATGAGGACAAATATC | 1200 |
|  | N1-N-12051 | GATTTTGCTGAAGGTGAATCAGA |  |
| 13 | SPB-11876 | CGAGGTAAAGTACCACGTACTCA | 750 |
|  | SPB-12595 | GTTGGATTTCTAACTTTATTRGARCG |  |
| T15 | N1-J-11891 | ATCCTCCTCTTCTATATTCAAC | 950 |
|  | 16S-N-12855 | GATTGCGACCTCGATGTTGG |  |
| 14 | SPB-12261 | TACCTCATAAGAAATAGTTTGAGC | 750 |
|  | SPB-13000 | TTACCTTAGGGATAACAGCGTAA |  |
| T16 | LR-J-12883 | CACCGGTTTGAACTCAGATC | 550 |
|  | LR-N-13398 | CGCCTGTTTATCAAAAACAT |  |
| T17 | LR-J-12888 | ACGCTGTTATCCCTAAAGTA | 1500 |
|  | SR-N-14373 | AATCCACGATGTACCTTACT |  |
| T18 | SR-J-14233 | AAGAGCGACGGGCGATGTGT | 550 |
|  | SR-N-14756 | GACAAAATTCGTGCCAGCAGT |  |
| T19 | SR-J-14612 | AGGGTATCTAATCCTAGTTT | 300 |
|  | SR-N-14922 | AAGTTTTATTTTGGCTTA |  |

Table S3. Organization of four newly sequenced Culicomorpha mt genomes.

Organization of the *Chaoborus* sp. mt genome

| Gene | Direction | Location | Size  (bp) | Anticodon | Codon |  | Intergenic nucleotide* |
| --- | --- | --- | --- | --- | --- | --- | --- |
|  |  |  |  |  | Start | Stop |  |
| *tRNA^Gln^* | J | 1-69 | 69 | TTG |  |  |  |
| *tRNA^Met^* | J | 141-209 | 69 | CAT |  |  | 71 |
| *ND2* | J | 210-1241 | 1032 |  | ATT | TAA | 0 |
| *tRNA^Trp^* | J | 1256-1325 | 70 | TCA |  |  | 14 |
| *tRNA^Cys^* | N | 1318-1384 | 67 | GCA |  |  | -8 |
| *tRNA^Tyr^* | N | 1389-1455 | 67 | GTA |  |  | 4 |
| *COI* | J | 1454-2989 | 1536 |  | CCG | TAG | -2 |
| *tRNA^Leu(UUR)^* | J | 3001-3066 | 66 | TAA |  |  | 11 |
| *COII* | J | 3070-3753 | 684 |  | ATG | TAA | 3 |
| *tRNA^Lys^* | J | 3756-3826 | 71 | CTT |  |  | 2 |
| *tRNA^Asp^* | J | 3831-3901 | 71 | GTC |  |  | 4 |
| *ATP8* | J | 3902-4063 | 162 |  | ATC | TAA | 0 |
| *ATP6* | J | 4057-4725 | 669 |  | ATG | TAA | -7 |
| *COIII* | J | 4737-5525 | 789 |  | ATG | TAA | 11 |
| *tRNA^Gly^* | J | 5529-5591 | 63 | TCC |  |  | 3 |
| *ND3* | J | 5592-5945 | 354 |  | ATT | TAA | 0 |
| *tRNA^Ala^* | J | 5956-6023 | 68 | TGC |  |  | 10 |
| *tRNA^Arg^* | J | 6023-6087 | 65 | TCG |  |  | -1 |
| *tRNA^Asn^* | J | 6097-6165 | 69 | GTT |  |  | 9 |
| *tRNA^Ser(AGN)^* | J | 6166-6232 | 67 | GCT |  |  | 0 |
| *tRNA^Glu^* | J | 6233-6303 | 71 | TTC |  |  | 0 |
| *tRNA^Phe^* | N | 6326-6391 | 66 | GAA |  |  | 22 |
| *ND5* | N | 6405-8153 | 1749 |  | ATT | TAA | 13 |
| *tRNA^His^* | N | 8151-8219 | 69 | GTG |  |  | -3 |
| *ND4* | N | 8226-9554 | 1329 |  | ATG | TAA | 6 |
| *ND4L* | N | 9548-9841 | 294 |  | ATG | TAA | -7 |
| *tRNA^Thr^* | J | 9844-9907 | 64 | TGT |  |  | 2 |
| *tRNA^Pro^* | N | 9908-9973 | 66 | TGG |  |  | 0 |
| *ND6* | J | 9976-10497 | 522 |  | ATT | TAA | 2 |
| *CytB* | J | 10519-11655 | 1137 |  | ATG | TAA | 21 |
| *tRNA^Ser(UCN)^* | J | 11655-11723 | 69 | TGA |  |  | -1 |
| *ND1* | N | 11741-12688 | 948 |  | ATA | TAA | 17 |
| *tRNA^Leu(CUN)^* | N | 12693-12758 | 66 | TAG |  |  | 4 |
| *lrRNA* | N | 12759-14109 | 1351 |  |  |  | 0 |
| *tRNA^Val^* | N | 14110-14181 | 72 | TAC |  |  | 0 |
| *srRNA* | N | 14182-14746 | 565 |  |  |  | 0 |

Intergenic nucleotide: minus indicates overlapping between genes.

Organization of the *Corethrella condita* mt genome

| Gene | Direction | Location | Size  (bp) | Anticodon | Codon |  | Intergenic nucleotide* |
| --- | --- | --- | --- | --- | --- | --- | --- |
|  |  |  |  |  | Start | Stop |  |
| *tRNA^Met^* | J | 1-69 | 69 | CAT |  |  |  |
| *ND2* | J | 70-1095 | 1026 |  | ATA | TAA | 0 |
| *tRNA^Trp^* | J | 1098-1164 | 67 | TCA |  |  | 2 |
| *tRNA^Cys^* | N | 1157-1222 | 66 | GCA |  |  | -8 |
| *tRNA^Tyr^* | N | 1227-1289 | 63 | GTA |  |  | 4 |
| *COI* | J | 1288-2826 | 1539 |  | TCG | TAA | -2 |
| *tRNA^Leu(UUR)^* | J | 2822-2885 | 64 | TAA |  |  | -5 |
| *COII* | J | 2886-3570 | 685 |  | GTG | T-tRNA | 0 |
| *tRNA^Lys^* | J | 3560-3638 | 71 | CTT |  |  | -11 |
| *tRNA^Asp^* | J | 3684-3752 | 69 | GTC |  |  | 45 |
| *ATP8* | J | 3753-3914 | 162 |  | ATT | TAA | 0 |
| *ATP6* | J | 3908-4585 | 678 |  | ATG | TAA | -7 |
| *COIII* | J | 4585-5373 | 789 |  | ATG | TAA | -1 |
| *tRNA^Gly^* | J | 5374-5438 | 65 | TCC |  |  | 0 |
| *ND3* | J | 5439-5792 | 354 |  | ATT | TAA | 0 |
| *tRNA^Ala^* | J | 5795-5861 | 67 | TGC |  |  | 2 |
| *tRNA^Arg^* | J | 5864-5927 | 64 | TCG |  |  | 2 |
| *tRNA^Asn^* | J | 5928-5993 | 66 | GTT |  |  | 0 |
| *tRNA^Ser(AGN)^* | J | 5994-6060 | 67 | GCT |  |  | 0 |
| *tRNA^Glu^* | J | 6062-6126 | 65 | TTC |  |  | 1 |
| *tRNA^Phe^* | N | 6139-6203 | 65 | GAA |  |  | 12 |
| *ND5* | N | 6203-7942 | 1740 |  | ATT | TAA | -1 |
| *tRNA^His^* | N | 7940-8003 | 64 | GTG |  |  | -3 |
| *ND4* | N | 8004-9342 | 1339 |  | ATG | T-tRNA | 0 |
| *ND4L* | N | 9336-9629 | 294 |  | ATG | TAA | -7 |
| *tRNA^Thr^* | J | 9632-9696 | 65 | TGT |  |  | 2 |
| *tRNA^Pro^* | N | 9697-9762 | 66 | TGG |  |  | 0 |
| *ND6* | J | 9765-10280 | 516 |  | ATT | TAA | 2 |
| *CytB* | J | 10280-11416 | 1137 |  | ATG | TAA | -1 |
| *tRNA^Ser(UCN)^* | J | 11418-11484 | 67 | TGA |  |  | 1 |
| *ND1* | N | 11501-12454 | 954 |  | TTG | TAA | 16 |
| *tRNA^Leu(CUN)^* | N | 12458-12519 | 62 | TAG |  |  | 3 |
| *lrRNA* | N | 12520-13850 | 1331 |  |  |  | 0 |
| *tRNA^Val^* | N | 13851-13917 | 67 | TAC |  |  | 0 |
| *srRNA* | N | 13918-14520 | 603 |  |  |  | 0 |

Intergenic nucleotide: minus indicates overlapping between genes.

Organization of the *Simulium quinquestriatum* mt genome

| Gene | Direction | Location | Size  (bp) | Anticodon | Codon |  | Intergenic nucleotide* |
| --- | --- | --- | --- | --- | --- | --- | --- |
|  |  |  |  |  | Start | Stop |  |
| *tRNA^Gln^* | N | 1-69 | 69 | TTG |  |  |  |
| *tRNA^Met^* | J | 115-183 | 69 | CAT |  |  | 45 |
| *ND2* | J | 184-1215 | 1032 |  | ATC | TAA | 0 |
| *tRNA^Trp^* | J | 1234-1304 | 71 | TCA |  |  | 18 |
| *tRNA^Cys^* | N | 1297-1363 | 67 | GCA |  |  | -8 |
| *tRNA^Tyr^* | N | 1376-1440 | 65 | GTA |  |  | 12 |
| *COI* | J | 1448-2986 | 1539 |  | TTG | TAA | 7 |
| *tRNA^Leu(UUR)^* | J | 2982-3047 | 66 | TAA |  |  | -5 |
| *COII* | J | 3062-3751 | 690 |  | ATG | TAA | 14 |
| *tRNA^Lys^* | J | 3754-3824 | 71 | CTT |  |  | 2 |
| *tRNA^Asp^* | J | 3843-3910 | 68 | GTC |  |  | 18 |
| *ATP8* | J | 3911-4081 | 171 |  | ATT | TAA | 0 |
| *ATP6* | J | 4075-4752 | 678 |  | ATG | TAA | -7 |
| *COIII* | J | 4758-5546 | 789 |  | ATG | TAA | 5 |
| *tRNA^Gly^* | J | 5549-5613 | 65 | TCC |  |  | 2 |
| *ND3* | J | 5614-5967 | 354 |  | ATT | TAA | 0 |
| *tRNA^Ala^* | J | 5987-6051 | 65 | TGC |  |  | 19 |
| *tRNA^Arg^* | J | 6060-6123 | 64 | TCG |  |  | 8 |
| *tRNA^Asn^* | J | 6124-6188 | 65 | GTT |  |  | 0 |
| *tRNA^Ser(AGN)^* | J | 6188-6255 | 68 | GCT |  |  | -1 |
| *tRNA^Glu^* | J | 6256-6322 | 67 | TTC |  |  | 0 |
| *tRNA^Phe^* | N | 6337-6402 | 66 | GAA |  |  | 14 |
| *ND5* | N | 6403-8139 | 1732 |  | ATT | TAA | 0 |
| *tRNA^His^* | N | 8140-8205 | 66 | GTG |  |  | 0 |
| *ND4* | N | 8209-9549 | 1341 |  | ATG | TAA | 3 |
| *ND4L* | N | 9543-9839 | 297 |  | ATG | TAA | -7 |
| *tRNA^Thr^* | J | 9842-9908 | 67 | TGT |  |  | 2 |
| *tRNA^Pro^* | N | 9909-9974 | 66 | TGG |  |  | 0 |
| *ND6* | J | 9968-10501 | 534 |  | ATT | TAA | -7 |
| *CytB* | J | 10501-11637 | 1137 |  | ATG | TAA | -1 |
| *tRNA^Ser(UCN)^* | J | 11641-11707 | 67 | TGA |  |  | 3 |
| *ND1* | N | 11750-12701 | 952 |  | ATA | T-tRNA | 42 |
| *tRNA^Leu(CUN)^* | N | 12696-12761 | 66 | TAG |  |  | -5 |
| *lrRNA* | N | 12762-14099 | 1338 |  |  |  | 0 |
| *tRNA^Val^* | N | 14100-14171 | 72 | TAC |  |  | 0 |
| *srRNA* | N | 14172-14737 | 566 |  |  |  | 0 |

Intergenic nucleotide: minus indicates overlapping between genes.

Organization of the *Thaumalea* sp. mt genome

| Gene | Direction | Location | Size  (bp) | Anticodon | Codon |  | Intergenic nucleotide* |
| --- | --- | --- | --- | --- | --- | --- | --- |
|  |  |  |  |  | Start | Stop |  |
| *tRNA^Met^* | J | 1-69 | 69 | CAT |  |  |  |
| *ND2* | J | 70-1101 | 1032 |  | ATG | TAA | 0 |
| *tRNA^Trp^* | J | 1100-1167 | 68 | TCA |  |  | -2 |
| *tRNA^Cys^* | N | 1160-1230 | 71 | GCA |  |  | -8 |
| *tRNA^Tyr^* | N | 1273-1338 | 66 | GTA |  |  | 42 |
| *COI* | J | 1341-2879 | 1539 |  | ACG | TAA | 2 |
| *tRNA^Leu(UUR)^* | J | 2875-2940 | 66 | TAA |  |  | -5 |
| *COII* | J | 2968-3655 | 688 |  | ATG | T-tRNA | 27 |
| *tRNA^Lys^* | J | 3656-3726 | 71 | CTT |  |  | 0 |
| *tRNA^Asp^* | J | 3726-3793 | 68 | GTC |  |  | -1 |
| *ATP8* | J | 3794-3961 | 168 |  | ATC | TAA | 0 |
| *ATP6* | J | 3955-4632 | 678 |  | ATG | TAA | -7 |
| *COIII* | J | 4632-5420 | 789 |  | ATG | TAA | -1 |
| *tRNA^Gly^* | J | 5420-5484 | 65 | TCC |  |  | -1 |
| *ND3* | J | 5476-5838 | 363 |  | ATA | TAG | -9 |
| *tRNA^Ala^* | J | 5837-5903 | 67 | TGC |  |  | -2 |
| *tRNA^Arg^* | J | 5903-5966 | 64 | TCG |  |  | -1 |
| *tRNA^Asn^* | J | 5969-6036 | 68 | GTT |  |  | 2 |
| *tRNA^Ser(AGN)^* | J | 6036-6104 | 69 | GCT |  |  | -1 |
| *tRNA^Glu^* | J | 6112-6177 | 66 | TTC |  |  | 7 |
| *tRNA^Phe^* | N | 6200-6266 | 67 | GAA |  |  | 22 |
| *ND5* | N | 6267-7998 | 1732 |  | ATG | T-tRNA | 0 |
| *tRNA^His^* | N | 7999-8063 | 65 | GTG |  |  | 0 |
| *ND4* | N | 8063-9403 | 1341 |  | ATG | TAA | -1 |
| *ND4L* | N | 9397-9693 | 297 |  | ATG | TAA | -7 |
| *tRNA^Thr^* | J | 9696-9762 | 67 | TGT |  |  | 2 |
| *tRNA^Pro^* | N | 9763-9828 | 66 | TGG |  |  | 0 |
| *ND6* | J | 9831-10355 | 525 |  | ATT | TAA | 2 |
| *CytB* | J | 10360-11496 | 1137 |  | ATG | TAG | 4 |
| *tRNA^Ser(UCN)^* | J | 11495-11563 | 69 | TGA |  |  | -2 |
| *ND1* | N | 11581-12531 | 951 |  | TTG | TAG | 17 |
| *tRNA^Leu(CUN)^* | N | 12533-12598 | 66 | TAG |  |  | 1 |
| *lrRNA* | N | 12599-13929 | 1331 |  |  |  | 0 |
| *tRNA^Val^* | N | 13930-13999 | 70 | TAC |  |  | 0 |
| *srRNA* | N | 14000-14610 | 611 |  |  |  | 0 |

Intergenic nucleotide: minus indicates overlapping between genes.

Table S4. Nucleotide composition of four newly sequenced Culicomorpha mt genomes.

Nucleotide composition of the *Chaoborus* sp. mt genome

| Feature | A% | T% | C% | G% | A+T% | C+G% | AT-skew | GC-skew |
| --- | --- | --- | --- | --- | --- | --- | --- | --- |
| Whole mitgenome | 40.1 | 36.7 | 14.5 | 8.8 | 76.8 | 23.2 | 0.04 | -0.25 |
| PCGs(J) | 32.8 | 40.4 | 16.0 | 10.8 | 73.2 | 26.8 | -0.10 | -0.19 |
| 1stcondon position(J) | 34.1 | 33 | 15.1 | 17.4 | 67.5 | 32.5 | 0.01 | 0.07 |
| 2ndcondon position(J) | 20.5 | 46 | 21.0 | 12.8 | 66.2 | 33.8 | -0.38 | -0.24 |
| 3rdcondon position(J) | 43.8 | 42 | 11.9 | 2.2 | 85.9 | 14.1 | 0.02 | -0.69 |
| PCGs(N) | 28.3 | 48.9 | 7.7 | 15.1 | 77.2 | 22.8 | -0.27 | 0.33 |
| 1stcondon position(N) | 28.7 | 44 | 7.7 | 19.9 | 72.4 | 27.6 | -0.21 | 0.44 |
| 2ndcondon position(N) | 18.5 | 52 | 13.8 | 15.8 | 70.4 | 29.6 | -0.48 | 0.07 |
| 3rdcondon position(N) | 37.7 | 51 | 1.5 | 9.7 | 88.8 | 11.3 | -0.15 | 0.73 |
| PCGs(All) | 31.1 | 43.7 | 12.8 | 12.5 | 74.8 | 25.2 | -0.17 | -0.01 |
| 1stcondon position(All) | 32.0 | 37 | 12.2 | 18.3 | 69.4 | 30.6 | -0.08 | 0.20 |
| 2ndcondon position(All) | 19.7 | 48 | 18.2 | 14.0 | 67.8 | 32.2 | -0.42 | -0.13 |
| 3rdcondon position(All) | 41.4 | 46 | 7.9 | 5.1 | 87.0 | 13.0 | -0.05 | -0.22 |
| tRNA genes | 42.1 | 39.8 | 7.7 | 10.4 | 81.9 | 18.1 | 0.03 | 0.15 |
| lrRNA | 38.3 | 45.8 | 5.2 | 10.7 | 84.2 | 15.8 | -0.09 | 0.35 |
| srRNA | 39.1 | 42.3 | 6.9 | 11.7 | 81.4 | 18.6 | -0.04 | 0.26 |

Note: AT-skew = (A-T)/(A+T)；GC-skew = (G-C)/(G +C)

Nucleotide composition of the *Corethrella condita* mt genome

| Feature | A% | T% | C% | G% | A+T% | C+G% | AT-skew | GC-skew |
| --- | --- | --- | --- | --- | --- | --- | --- | --- |
| Whole mitgenome | 38.9 | 36.5 | 14.7 | 9.9 | 75.4 | 24.6 | 0.03 | -0.19 |
| PCGs(J) | 31.9 | 40.7 | 15.3 | 12.2 | 72.5 | 27.5 | -0.12 | -0.11 |
| 1stcondon position(J) | 32.0 | 34 | 14.2 | 19.7 | 66.1 | 33.9 | -0.03 | 0.16 |
| 2ndcondon position(J) | 21.1 | 44 | 21.7 | 12.7 | 65.6 | 34.4 | -0.36 | -0.26 |
| 3rdcondon position(J) | 42.5 | 43 | 9.9 | 4.1 | 86.0 | 14.0 | -0.01 | -0.42 |
| PCGs(N) | 28.2 | 46.9 | 8.6 | 16.4 | 75.1 | 24.9 | -0.25 | 0.31 |
| 1stcondon position(N) | 28.6 | 43 | 7.4 | 21.2 | 71.4 | 28.6 | -0.20 | 0.49 |
| 2ndcondon position(N) | 18.7 | 49 | 15.1 | 16.8 | 68.1 | 31.9 | -0.45 | 0.05 |
| 3rdcondon position(N) | 37.2 | 48 | 3.2 | 11.2 | 85.6 | 14.4 | -0.13 | 0.56 |
| PCGs(All) | 30.5 | 43.1 | 12.7 | 13.8 | 73.5 | 26.5 | -0.17 | 0.04 |
| 1stcondon position(All) | 30.7 | 37 | 11.5 | 20.3 | 68.2 | 31.8 | -0.10 | 0.28 |
| 2ndcondon position(All) | 20.2 | 46 | 19.2 | 14.3 | 66.6 | 33.4 | -0.39 | -0.15 |
| 3rdcondon position(All) | 40.5 | 45 | 7.3 | 6.8 | 85.8 | 14.2 | -0.06 | -0.04 |
| tRNA genes | 40.1 | 39.0 | 8.9 | 11.9 | 79.2 | 20.8 | 0.01 | 0.14 |
| lrRNA | 40.3 | 43.7 | 5.2 | 10.9 | 83.9 | 16.1 | -0.04 | 0.36 |
| srRNA | 38.1 | 42.0 | 6.8 | 13.1 | 80.1 | 19.9 | -0.05 | 0.32 |

Note: AT-skew = (A-T)/(A+T)；GC-skew = (G-C)/(G +C)

Nucleotide composition of the *Simulium quinquestriatum* mt genome

| Feature | A% | T% | C% | G% | A+T% | C+G% | AT-skew | GC-skew |
| --- | --- | --- | --- | --- | --- | --- | --- | --- |
| Whole mitgenome | 39.1 | 36.1 | 14.6 | 10.3 | 75.2 | 24.8 | 0.04 | -0.17 |
| PCGs(J) | 32.2 | 40.1 | 15.5 | 12.2 | 72.3 | 27.7 | -0.11 | -0.12 |
| 1stcondon position(J) | 31.7 | 33 | 14.3 | 20.7 | 65.0 | 35.0 | -0.03 | 0.18 |
| 2ndcondon position(J) | 20.8 | 43 | 22.7 | 13.5 | 63.8 | 36.2 | -0.35 | -0.25 |
| 3rdcondon position(J) | 44.2 | 44 | 9.4 | 2.5 | 88.2 | 11.8 | 0.00 | -0.58 |
| PCGs(N) | 28.8 | 47.5 | 9.1 | 14.6 | 76.3 | 23.7 | -0.25 | 0.23 |
| 1stcondon position(N) | 29.8 | 42 | 8.3 | 19.8 | 71.9 | 28.1 | -0.17 | 0.41 |
| 2ndcondon position(N) | 19.1 | 48 | 16.8 | 15.6 | 67.6 | 32.4 | -0.43 | -0.04 |
| 3rdcondon position(N) | 37.5 | 52 | 2.2 | 8.4 | 89.4 | 10.6 | -0.16 | 0.58 |
| PCGs(All) | 30.9 | 43.0 | 13.0 | 13.1 | 73.9 | 26.1 | -0.16 | 0.00 |
| 1stcondon position(All) | 30.9 | 37 | 12.0 | 20.3 | 67.7 | 32.3 | -0.09 | 0.26 |
| 2ndcondon position(All) | 20.2 | 45 | 20.4 | 14.3 | 65.3 | 34.7 | -0.38 | -0.18 |
| 3rdcondon position(All) | 41.6 | 47 | 6.6 | 4.7 | 88.6 | 11.4 | -0.06 | -0.16 |
| tRNA genes | 38.0 | 38.7 | 10.1 | 13.2 | 76.7 | 23.3 | -0.01 | 0.13 |
| lrRNA | 36.7 | 45.5 | 6.0 | 11.8 | 82.2 | 17.8 | -0.11 | 0.33 |
| srRNA | 36.9 | 38.5 | 9.7 | 14.8 | 75.4 | 24.6 | -0.02 | 0.21 |

Note: AT-skew = (A-T)/(A+T)；GC-skew = (G-C)/(G +C)

Nucleotide composition of the *Thaumalea* sp. mt genome

| Feature | A% | T% | C% | G% | A+T% | C+G% | AT-skew | GC-skew |
| --- | --- | --- | --- | --- | --- | --- | --- | --- |
| Whole mitgenome | 36.2 | 33.0 | 18.7 | 12.1 | 69.2 | 30.8 | 0.05 | -0.21 |
| PCGs(J) | 29.4 | 36.5 | 20.4 | 13.7 | 65.9 | 34.1 | -0.11 | -0.20 |
| 1stcondon position(J) | 30.8 | 30 | 17.5 | 21.4 | 61.1 | 38.9 | 0.01 | 0.10 |
| 2ndcondon position(J) | 20.3 | 44 | 23.1 | 13.1 | 63.9 | 36.1 | -0.36 | -0.28 |
| 3rdcondon position(J) | 37.0 | 36 | 20.6 | 6.7 | 72.8 | 27.2 | 0.02 | -0.51 |
| PCGs(N) | 24.1 | 43.9 | 12.3 | 19.7 | 68.0 | 32.0 | -0.29 | 0.23 |
| 1stcondon position(N) | 28.8 | 40 | 10.1 | 21.3 | 68.7 | 31.3 | -0.16 | 0.36 |
| 2ndcondon position(N) | 18.1 | 48 | 16.9 | 16.9 | 66.2 | 33.8 | -0.45 | 0.00 |
| 3rdcondon position(N) | 25.4 | 44 | 9.9 | 20.8 | 69.2 | 30.8 | -0.27 | 0.35 |
| PCGs(All) | 27.4 | 39.4 | 17.3 | 16.0 | 66.7 | 33.3 | -0.18 | -0.04 |
| 1stcondon position(All) | 30.1 | 34 | 14.7 | 21.4 | 64.0 | 36.0 | -0.06 | 0.19 |
| 2ndcondon position(All) | 19.5 | 45 | 20.7 | 14.5 | 64.8 | 35.2 | -0.40 | -0.18 |
| 3rdcondon position(All) | 32.5 | 39 | 16.5 | 12.1 | 71.4 | 28.6 | -0.09 | -0.15 |
| tRNA genes | 37.8 | 38.4 | 10.4 | 13.4 | 76.2 | 23.8 | -0.01 | 0.13 |
| lrRNA | 37.3 | 42.9 | 6.4 | 13.4 | 80.2 | 19.8 | -0.07 | 0.35 |
| srRNA | 36.2 | 38.3 | 10.3 | 15.2 | 74.5 | 25.5 | -0.03 | 0.19 |

Note: AT-skew = (A-T)/(A+T)；GC-skew = (G-C)/(G +C)

Table S5. Codon usage of four newly sequenced Culicomorpha mt genomes.

Codon usage of the *Chaoborus* sp. mt genome

| Amino acid | Codon | N | RSCU | N+ | RSCU | N- | RSCU |
| --- | --- | --- | --- | --- | --- | --- | --- |
| Phe(F) | **UUU(F)** | 359 | 1.84 | 190 | 1.72 | 169 | 1.99 |
|  | UUC(F) | 32 | 0.16 | 31 | 0.28 | 1 | 0.01 |
| Leu^UUR^ (L) | **UUA(L)** | 440 | 4.38 | 217 | 3.98 | 223 | 4.85 |
|  | UUG(L) | 38 | 0.38 | 7 | 0.13 | 31 | 0.67 |
| Leu^CUN^ (L) | **CUU(L)** | 64 | 0.64 | 46 | 0.84 | 18 | 0.39 |
|  | CUC(L) | 3 | 0.03 | 3 | 0.06 | 0 | 0 |
|  | CUA(L) | 51 | 0.51 | 49 | 0.9 | 2 | 0.04 |
|  | CUG(L) | 7 | 0.07 | 5 | 0.09 | 2 | 0.04 |
| Ile (I) | **AUU(I)** | 341 | 1.78 | 234 | 1.71 | 107 | 1.96 |
|  | AUC(I) | 42 | 0.22 | 40 | 0.29 | 2 | 0.04 |
| Met (M) | **AUA(M)** | 226 | 1.83 | 137 | 1.9 | 89 | 1.73 |
|  | AUG(M) | 21 | 0.17 | 7 | 0.1 | 14 | 0.27 |
| Val (V) | **GUU(V)** | 88 | 2.02 | 27 | 1.29 | 61 | 2.71 |
|  | GUC(V) | 10 | 0.23 | 7 | 0.33 | 3 | 0.13 |
|  | GUA(V) | 70 | 1.61 | 48 | 2.29 | 22 | 0.98 |
|  | GUG(V) | 6 | 0.14 | 2 | 0.1 | 4 | 0.18 |
| Ser^UCN^ (S) | **UCU(S)** | 118 | 2.8 | 66 | 2.72 | 52 | 2.91 |
|  | UCC(S) | 16 | 0.38 | 15 | 0.62 | 1 | 0.06 |
|  | UCA(S) | 85 | 2.02 | 64 | 2.64 | 21 | 1.17 |
|  | UCG(S) | 3 | 0.07 | 1 | 0.04 | 2 | 0.11 |
| Pro (P) | **CCU(P)** | 75 | 2.27 | 46 | 1.84 | 29 | 3.63 |
|  | CCC(P) | 18 | 0.55 | 18 | 0.72 | 0 | 0 |
|  | CCA(P) | 37 | 1.12 | 34 | 1.36 | 3 | 0.38 |
|  | CCG(P) | 2 | 0.06 | 2 | 0.08 | 0 | 0 |
| Thr (T) | ACU(T) | 82 | 1.94 | 56 | 1.66 | 26 | 3.06 |
|  | ACC(T) | 25 | 0.59 | 25 | 0.74 | 0 | 0 |
|  | **ACA(T)** | 60 | 1.42 | 52 | 1.54 | 8 | 0.94 |
|  | ACG(T) | 2 | 0.05 | 2 | 0.06 | 0 | 0 |
| Ala (A) | **GCU(A)** | 87 | 2.23 | 45 | 1.8 | 42 | 3 |
|  | GCC(A) | 22 | 0.56 | 21 | 0.84 | 1 | 0.07 |
|  | GCA(A) | 46 | 1.18 | 33 | 1.32 | 13 | 0.93 |
|  | GCG(A) | 1 | 0.03 | 1 | 0.04 | 0 | 0 |
| Tyr (Y) | **UAU(Y)** | 115 | 1.47 | 46 | 1.1 | 69 | 1.92 |
|  | UAC(Y) | 41 | 0.53 | 38 | 0.9 | 3 | 0.08 |
| Stop (*) | **UAA(*)** | 13 | 2 | 9 | 2 | 4 | 2 |
|  | UAG(*) | 0 | 0 | 0 | 0 | 0 | 0 |
| His (H) | **CAU(H)** | 58 | 1.61 | 47 | 1.57 | 11 | 1.83 |
|  | CAC(H) | 14 | 0.39 | 13 | 0.43 | 1 | 0.17 |
| Gln (Q) | CAA(Q) | 65 | 1.78 | 48 | 2 | 17 | 1.36 |
|  | CAG(Q) | 8 | 0.22 | 0 | 0 | 8 | 0.64 |
| Asn (N) | **AAU(N)** | 149 | 1.63 | 97 | 1.52 | 52 | 1.89 |
|  | AAC(N) | 34 | 0.37 | 31 | 0.48 | 3 | 0.11 |
| Lys (K) | **AAA(K)** | 87 | 1.76 | 53 | 1.96 | 34 | 1.51 |
|  | AAG(K) | 12 | 0.24 | 1 | 0.04 | 11 | 0.49 |
| Asp (D) | **GAU(D)** | 53 | 1.56 | 28 | 1.3 | 25 | 2 |
|  | GAC(D) | 15 | 0.44 | 15 | 0.7 | 0 | 0 |
| Glu (E) | **GAA(E)** | 63 | 1.75 | 43 | 1.95 | 20 | 1.43 |
|  | GAG(E) | 9 | 0.25 | 1 | 0.05 | 8 | 0.57 |
| Cys (C) | **UGU(C)** | 32 | 1.6 | 7 | 1.08 | 25 | 1.85 |
|  | UGC(C) | 8 | 0.4 | 6 | 0.92 | 2 | 0.15 |
| Trp (W) | **UGA(W)** | 84 | 1.73 | 67 | 1.91 | 17 | 1.26 |
|  | UGG(W) | 13 | 0.27 | 3 | 0.09 | 10 | 0.74 |
| Arg (R) | CGU(R) | 13 | 0.95 | 5 | 0.57 | 8 | 1.6 |
|  | CGC(R) | 1 | 0.07 | 1 | 0.11 | 0 | 0 |
|  | **CGA(R)** | 36 | 2.62 | 29 | 3.31 | 7 | 1.4 |
|  | CGG(R) | 5 | 0.36 | 0 | 0 | 5 | 1 |
| Ser^AGN^ (S) | **AGU(S)** | 36 | 0.85 | 14 | 0.58 | 22 | 1.23 |
|  | AGC(S) | 7 | 0.17 | 3 | 0.12 | 4 | 0.22 |
|  | AGA(S) | 72 | 1.71 | 31 | 1.28 | 41 | 2.29 |
|  | AGG(S) | 0 | 0 | 0 | 0 | 0 | 0 |
| Gly (G) | GGU(G) | 31 | 0.58 | 12 | 0.38 | 19 | 0.87 |
|  | GGC(G) | 8 | 0.15 | 7 | 0.22 | 1 | 0.05 |
|  | **GGA(G)** | 113 | 2.1 | 91 | 2.84 | 22 | 1.01 |
|  | GGG(G) | 63 | 1.17 | 18 | 0.56 | 45 | 2.07 |

Codon usage of the *Corethrella condita* mt genome

| Amino acid | Codon | N | RSCU | N+ | RSCU | N- | RSCU |
| --- | --- | --- | --- | --- | --- | --- | --- |
| Phe(F) | **UUU(F)** | 314 | 1.82 | 170 | 1.71 | 144 | 1.96 |
|  | UUC(F) | 32 | 0.18 | 29 | 0.29 | 3 | 0.04 |
| Leu^UUR^ (L) | **UUA(L)** | 456 | 4.46 | 245 | 4.39 | 211 | 4.55 |
|  | UUG(L) | 64 | 0.63 | 17 | 0.3 | 47 | 1.01 |
| Leu^CUN^ (L) | **CUU(L)** | 42 | 0.41 | 33 | 0.59 | 9 | 0.19 |
|  | CUC(L) | 4 | 0.04 | 4 | 0.07 | 0 | 0 |
|  | CUA(L) | 43 | 0.42 | 33 | 0.59 | 10 | 0.22 |
|  | CUG(L) | 4 | 0.04 | 3 | 0.05 | 1 | 0.02 |
| Ile (I) | **AUU(I)** | 321 | 1.79 | 220 | 1.72 | 101 | 1.96 |
|  | AUC(I) | 38 | 0.21 | 36 | 0.28 | 2 | 0.04 |
| Met (M) | **AUA(M)** | 182 | 1.66 | 111 | 1.79 | 71 | 1.49 |
|  | AUG(M) | 37 | 0.34 | 13 | 0.21 | 24 | 0.51 |
| Val (V) | **GUU(V)** | 101 | 2.06 | 42 | 1.58 | 59 | 2.62 |
|  | GUC(V) | 5 | 0.1 | 4 | 0.15 | 1 | 0.04 |
|  | GUA(V) | 73 | 1.49 | 51 | 1.92 | 22 | 0.98 |
|  | GUG(V) | 17 | 0.35 | 9 | 0.34 | 8 | 0.36 |
| Ser^UCN^ (S) | **UCU(S)** | 122 | 2.93 | 60 | 2.58 | 62 | 3.37 |
|  | UCC(S) | 9 | 0.22 | 8 | 0.34 | 1 | 0.05 |
|  | UCA(S) | 83 | 1.99 | 70 | 3.01 | 13 | 0.71 |
|  | UCG(S) | 6 | 0.14 | 5 | 0.22 | 1 | 0.05 |
| Pro (P) | **CCU(P)** | 58 | 1.8 | 39 | 1.59 | 19 | 2.45 |
|  | CCC(P) | 31 | 0.96 | 25 | 1.02 | 6 | 0.77 |
|  | CCA(P) | 33 | 1.02 | 29 | 1.18 | 4 | 0.52 |
|  | CCG(P) | 7 | 0.22 | 5 | 0.2 | 2 | 0.26 |
| Thr (T) | ACU(T) | 83 | 1.85 | 56 | 1.68 | 27 | 2.35 |
|  | ACC(T) | 14 | 0.31 | 12 | 0.36 | 2 | 0.17 |
|  | **ACA(T)** | 77 | 1.72 | 62 | 1.86 | 15 | 1.3 |
|  | ACG(T) | 5 | 0.11 | 3 | 0.09 | 2 | 0.17 |
| Ala (A) | **GCU(A)** | 95 | 2.02 | 60 | 1.94 | 35 | 2.19 |
|  | GCC(A) | 28 | 0.6 | 20 | 0.65 | 8 | 0.5 |
|  | GCA(A) | 62 | 1.32 | 43 | 1.39 | 19 | 1.19 |
|  | GCG(A) | 3 | 0.06 | 1 | 0.03 | 2 | 0.13 |
| Tyr (Y) | **UAU(Y)** | 141 | 1.71 | 72 | 1.6 | 69 | 1.84 |
|  | UAC(Y) | 24 | 0.29 | 18 | 0.4 | 6 | 0.16 |
| Stop (*) | **UAA(*)** | 11 | 2 | 8 | 2 | 3 | 2 |
|  | UAG(*) | 0 | 0 | 0 | 0 | 0 | 0 |
| His (H) | **CAU(H)** | 60 | 1.56 | 49 | 1.51 | 11 | 1.83 |
|  | CAC(H) | 17 | 0.44 | 16 | 0.49 | 1 | 0.17 |
| Gln (Q) | CAA(Q) | 62 | 1.72 | 46 | 1.8 | 16 | 1.52 |
|  | CAG(Q) | 10 | 0.28 | 5 | 0.2 | 5 | 0.48 |
| Asn (N) | **AAU(N)** | 170 | 1.76 | 114 | 1.7 | 56 | 1.9 |
|  | AAC(N) | 23 | 0.24 | 20 | 0.3 | 3 | 0.1 |
| Lys (K) | **AAA(K)** | 73 | 1.72 | 42 | 1.87 | 31 | 1.55 |
|  | AAG(K) | 12 | 0.28 | 3 | 0.13 | 9 | 0.45 |
| Asp (D) | **GAU(D)** | 53 | 1.54 | 32 | 1.39 | 21 | 1.83 |
|  | GAC(D) | 16 | 0.46 | 14 | 0.61 | 2 | 0.17 |
| Glu (E) | **GAA(E)** | 66 | 1.61 | 41 | 1.78 | 25 | 1.39 |
|  | GAG(E) | 16 | 0.39 | 5 | 0.22 | 11 | 0.61 |
| Cys (C) | **UGU(C)** | 31 | 1.68 | 7 | 1.08 | 24 | 2 |
|  | UGC(C) | 6 | 0.32 | 6 | 0.92 | 0 | 0 |
| Trp (W) | **UGA(W)** | 94 | 1.88 | 65 | 1.94 | 29 | 1.76 |
|  | UGG(W) | 6 | 0.12 | 2 | 0.06 | 4 | 0.24 |
| Arg (R) | CGU(R) | 12 | 0.8 | 6 | 0.63 | 6 | 1.09 |
|  | CGC(R) | 0 | 0 | 0 | 0 | 0 | 0 |
|  | **CGA(R)** | 39 | 2.6 | 27 | 2.84 | 12 | 2.18 |
|  | CGG(R) | 9 | 0.6 | 5 | 0.53 | 4 | 0.73 |
| Ser^AGN^ (S) | **AGU(S)** | 49 | 1.18 | 18 | 0.77 | 31 | 1.69 |
|  | AGC(S) | 9 | 0.22 | 4 | 0.17 | 5 | 0.27 |
|  | AGA(S) | 55 | 1.32 | 21 | 0.9 | 34 | 1.85 |
|  | AGG(S) | 0 | 0 | 0 | 0 | 0 | 0 |
| Gly (G) | GGU(G) | 44 | 0.79 | 20 | 0.61 | 24 | 1.03 |
|  | GGC(G) | 18 | 0.32 | 12 | 0.37 | 6 | 0.26 |
|  | **GGA(G)** | 103 | 1.84 | 81 | 2.47 | 22 | 0.95 |
|  | GGG(G) | 59 | 1.05 | 18 | 0.55 | 41 | 1.76 |

Codon usage of the *Simulium quinquestriatum* mt genome

| Amino acid | Codon | N | RSCU | N+ | RSCU | N- | RSCU |
| --- | --- | --- | --- | --- | --- | --- | --- |
| Phe(F) | **UUU(F)** | 322 | 1.83 | 182 | 1.76 | 140 | 1.94 |
|  | UUC(F) | 29 | 0.17 | 25 | 0.24 | 4 | 0.06 |
| Leu^UUR^ (L) | **UUA(L)** | 460 | 4.58 | 246 | 4.51 | 214 | 4.67 |
|  | UUG(L) | 46 | 0.46 | 10 | 0.18 | 36 | 0.79 |
| Leu^CUN^ (L) | **CUU(L)** | 57 | 0.57 | 39 | 0.72 | 18 | 0.39 |
|  | CUC(L) | 5 | 0.05 | 5 | 0.09 | 0 | 0 |
|  | CUA(L) | 30 | 0.3 | 26 | 0.48 | 4 | 0.09 |
|  | CUG(L) | 4 | 0.04 | 1 | 0.02 | 3 | 0.07 |
| Ile (I) | **AUU(I)** | 324 | 1.84 | 213 | 1.79 | 111 | 1.95 |
|  | AUC(I) | 28 | 0.16 | 25 | 0.21 | 3 | 0.05 |
| Met (M) | **AUA(M)** | 185 | 1.8 | 102 | 1.87 | 83 | 1.73 |
|  | AUG(M) | 20 | 0.2 | 7 | 0.13 | 13 | 0.27 |
| Val (V) | **GUU(V)** | 92 | 2.03 | 44 | 1.59 | 48 | 2.74 |
|  | GUC(V) | 5 | 0.11 | 3 | 0.11 | 2 | 0.11 |
|  | GUA(V) | 73 | 1.61 | 58 | 2.09 | 15 | 0.86 |
|  | GUG(V) | 11 | 0.24 | 6 | 0.22 | 5 | 0.29 |
| Ser^UCN^ (S) | **UCU(S)** | 126 | 2.99 | 62 | 2.67 | 64 | 3.39 |
|  | UCC(S) | 13 | 0.31 | 12 | 0.52 | 1 | 0.05 |
|  | UCA(S) | 76 | 1.8 | 56 | 2.41 | 20 | 1.06 |
|  | UCG(S) | 5 | 0.12 | 3 | 0.13 | 2 | 0.11 |
| Pro (P) | **CCU(P)** | 67 | 1.81 | 44 | 1.59 | 23 | 2.49 |
|  | CCC(P) | 16 | 0.43 | 15 | 0.54 | 1 | 0.11 |
|  | CCA(P) | 61 | 1.65 | 50 | 1.8 | 11 | 1.19 |
|  | CCG(P) | 4 | 0.11 | 2 | 0.07 | 2 | 0.22 |
| Thr (T) | ACU(T) | 93 | 1.9 | 60 | 1.6 | 33 | 2.87 |
|  | ACC(T) | 20 | 0.41 | 17 | 0.45 | 3 | 0.26 |
|  | **ACA(T)** | 81 | 1.65 | 71 | 1.89 | 10 | 0.87 |
|  | ACG(T) | 2 | 0.04 | 2 | 0.05 | 0 | 0 |
| Ala (A) | **GCU(A)** | 101 | 2.01 | 54 | 1.67 | 47 | 2.61 |
|  | GCC(A) | 31 | 0.62 | 24 | 0.74 | 7 | 0.39 |
|  | GCA(A) | 67 | 1.33 | 50 | 1.55 | 17 | 0.94 |
|  | GCG(A) | 2 | 0.04 | 1 | 0.03 | 1 | 0.06 |
| Tyr (Y) | **UAU(Y)** | 136 | 1.77 | 66 | 1.59 | 70 | 1.97 |
|  | UAC(Y) | 18 | 0.23 | 17 | 0.41 | 1 | 0.03 |
| Stop (*) | **UAA(*)** | 12 | 2 | 9 | 2 | 3 | 2 |
|  | UAG(*) | 0 | 0 | 0 | 0 | 0 | 0 |
| His (H) | **CAU(H)** | 62 | 1.59 | 46 | 1.48 | 16 | 2 |
|  | CAC(H) | 16 | 0.41 | 16 | 0.52 | 0 | 0 |
| Gln (Q) | CAA(Q) | 65 | 1.86 | 47 | 1.92 | 18 | 1.71 |
|  | CAG(Q) | 5 | 0.14 | 2 | 0.08 | 3 | 0.29 |
| Asn (N) | **AAU(N)** | 183 | 1.8 | 118 | 1.72 | 65 | 1.97 |
|  | AAC(N) | 20 | 0.2 | 19 | 0.28 | 1 | 0.03 |
| Lys (K) | **AAA(K)** | 76 | 1.75 | 44 | 2 | 32 | 1.49 |
|  | AAG(K) | 11 | 0.25 | 0 | 0 | 11 | 0.51 |
| Asp (D) | **GAU(D)** | 61 | 1.65 | 38 | 1.49 | 23 | 2 |
|  | GAC(D) | 13 | 0.35 | 13 | 0.51 | 0 | 0 |
| Glu (E) | **GAA(E)** | 71 | 1.8 | 45 | 1.96 | 26 | 1.58 |
|  | GAG(E) | 8 | 0.2 | 1 | 0.04 | 7 | 0.42 |
| Cys (C) | **UGU(C)** | 31 | 1.77 | 8 | 1.6 | 23 | 1.84 |
|  | UGC(C) | 4 | 0.23 | 2 | 0.4 | 2 | 0.16 |
| Trp (W) | **UGA(W)** | 92 | 1.86 | 68 | 1.92 | 24 | 1.71 |
|  | UGG(W) | 7 | 0.14 | 3 | 0.08 | 4 | 0.29 |
| Arg (R) | CGU(R) | 9 | 0.61 | 1 | 0.11 | 8 | 1.52 |
|  | CGC(R) | 2 | 0.14 | 1 | 0.11 | 1 | 0.19 |
|  | **CGA(R)** | 44 | 2.98 | 34 | 3.58 | 10 | 1.9 |
|  | CGG(R) | 4 | 0.27 | 2 | 0.21 | 2 | 0.38 |
| Ser^AGN^ (S) | **AGU(S)** | 54 | 1.28 | 19 | 0.82 | 35 | 1.85 |
|  | AGC(S) | 13 | 0.31 | 11 | 0.47 | 2 | 0.11 |
|  | AGA(S) | 48 | 1.14 | 22 | 0.95 | 26 | 1.38 |
|  | AGG(S) | 2 | 0.05 | 1 | 0.04 | 1 | 0.05 |
| Gly (G) | GGU(G) | 46 | 0.81 | 22 | 0.63 | 24 | 1.1 |
|  | GGC(G) | 15 | 0.26 | 11 | 0.31 | 4 | 0.18 |
|  | **GGA(G)** | 119 | 2.1 | 91 | 2.6 | 28 | 1.29 |
|  | GGG(G) | 47 | 0.83 | 16 | 0.46 | 31 | 1.43 |

Codon usage of the *Thaumalea* sp. mt genome

| Amino acid | Codon | N | RSCU | N+ | RSCU | N- | RSCU |
| --- | --- | --- | --- | --- | --- | --- | --- |
| Phe(F) | **UUU(F)** | 276 | 1.68 | 146 | 1.54 | 130 | 1.87 |
|  | UUC(F) | 52 | 0.32 | 43 | 0.46 | 9 | 0.13 |
| Leu^UUR^ (L) | **UUA(L)** | 320 | 3.12 | 182 | 3.15 | 138 | 3.09 |
|  | UUG(L) | 99 | 0.97 | 20 | 0.35 | 79 | 1.77 |
| Leu^CUN^ (L) | **CUU(L)** | 85 | 0.83 | 62 | 1.07 | 23 | 0.51 |
|  | CUC(L) | 16 | 0.16 | 13 | 0.22 | 3 | 0.07 |
|  | CUA(L) | 76 | 0.74 | 62 | 1.07 | 14 | 0.31 |
|  | CUG(L) | 19 | 0.19 | 8 | 0.14 | 11 | 0.25 |
| Ile (I) | **AUU(I)** | 258 | 1.58 | 162 | 1.48 | 96 | 1.79 |
|  | AUC(I) | 68 | 0.42 | 57 | 0.52 | 11 | 0.21 |
| Met (M) | **AUA(M)** | 146 | 1.34 | 97 | 1.6 | 49 | 1.01 |
|  | AUG(M) | 72 | 0.66 | 24 | 0.4 | 48 | 0.99 |
| Val (V) | **GUU(V)** | 94 | 1.8 | 41 | 1.28 | 53 | 2.62 |
|  | GUC(V) | 27 | 0.52 | 24 | 0.75 | 3 | 0.15 |
|  | GUA(V) | 66 | 1.26 | 55 | 1.72 | 11 | 0.54 |
|  | GUG(V) | 22 | 0.42 | 8 | 0.25 | 14 | 0.69 |
| Ser^UCN^ (S) | **UCU(S)** | 128 | 3.13 | 73 | 3.32 | 55 | 2.91 |
|  | UCC(S) | 24 | 0.59 | 16 | 0.73 | 8 | 0.42 |
|  | UCA(S) | 49 | 1.2 | 37 | 1.68 | 12 | 0.64 |
|  | UCG(S) | 15 | 0.37 | 4 | 0.18 | 11 | 0.58 |
| Pro (P) | **CCU(P)** | 44 | 1.26 | 26 | 1.02 | 18 | 1.89 |
|  | CCC(P) | 63 | 1.8 | 52 | 2.04 | 11 | 1.16 |
|  | CCA(P) | 24 | 0.69 | 19 | 0.75 | 5 | 0.53 |
|  | CCG(P) | 9 | 0.26 | 5 | 0.2 | 4 | 0.42 |
| Thr (T) | ACU(T) | 88 | 1.68 | 64 | 1.56 | 24 | 2.09 |
|  | ACC(T) | 47 | 0.9 | 37 | 0.9 | 10 | 0.87 |
|  | **ACA(T)** | 58 | 1.1 | 51 | 1.24 | 7 | 0.61 |
|  | ACG(T) | 17 | 0.32 | 12 | 0.29 | 5 | 0.43 |
| Ala (A) | **GCU(A)** | 56 | 1.07 | 35 | 1.03 | 21 | 1.14 |
|  | GCC(A) | 89 | 1.7 | 55 | 1.62 | 34 | 1.84 |
|  | GCA(A) | 41 | 0.78 | 37 | 1.09 | 4 | 0.22 |
|  | GCG(A) | 24 | 0.46 | 9 | 0.26 | 15 | 0.81 |
| Tyr (Y) | **UAU(Y)** | 114 | 1.44 | 48 | 1.12 | 66 | 1.83 |
|  | UAC(Y) | 44 | 0.56 | 38 | 0.88 | 6 | 0.17 |
| Stop (*) | **UAA(*)** | 8 | 1.45 | 6 | 1.5 | 2 | 1.33 |
|  | UAG(*) | 3 | 0.55 | 2 | 0.5 | 1 | 0.67 |
| His (H) | **CAU(H)** | 35 | 0.9 | 29 | 0.89 | 6 | 0.92 |
|  | CAC(H) | 43 | 1.1 | 36 | 1.11 | 7 | 1.08 |
| Gln (Q) | CAA(Q) | 62 | 1.63 | 51 | 1.85 | 11 | 1.05 |
|  | CAG(Q) | 14 | 0.37 | 4 | 0.15 | 10 | 0.95 |
| Asn (N) | **AAU(N)** | 136 | 1.55 | 81 | 1.4 | 55 | 1.83 |
|  | AAC(N) | 40 | 0.45 | 35 | 0.6 | 5 | 0.17 |
| Lys (K) | **AAA(K)** | 66 | 1.55 | 41 | 1.82 | 25 | 1.25 |
|  | AAG(K) | 19 | 0.45 | 4 | 0.18 | 15 | 0.75 |
| Asp (D) | **GAU(D)** | 35 | 1 | 22 | 0.9 | 13 | 1.24 |
|  | GAC(D) | 35 | 1 | 27 | 1.1 | 8 | 0.76 |
| Glu (E) | **GAA(E)** | 48 | 1.26 | 38 | 1.69 | 10 | 0.65 |
|  | GAG(E) | 28 | 0.74 | 7 | 0.31 | 21 | 1.35 |
| Cys (C) | **UGU(C)** | 31 | 1.59 | 8 | 1.23 | 23 | 1.77 |
|  | UGC(C) | 8 | 0.41 | 5 | 0.77 | 3 | 0.23 |
| Trp (W) | **UGA(W)** | 84 | 1.68 | 59 | 1.71 | 25 | 1.61 |
|  | UGG(W) | 16 | 0.32 | 10 | 0.29 | 6 | 0.39 |
| Arg (R) | CGU(R) | 11 | 0.75 | 5 | 0.54 | 6 | 1.09 |
|  | CGC(R) | 5 | 0.34 | 5 | 0.54 | 0 | 0 |
|  | **CGA(R)** | 30 | 2.03 | 24 | 2.59 | 6 | 1.09 |
|  | CGG(R) | 13 | 0.88 | 3 | 0.32 | 10 | 1.82 |
| Ser^AGN^ (S) | **AGU(S)** | 34 | 0.83 | 11 | 0.5 | 23 | 1.22 |
|  | AGC(S) | 20 | 0.49 | 11 | 0.5 | 9 | 0.48 |
|  | AGA(S) | 57 | 1.39 | 24 | 1.09 | 33 | 1.75 |
|  | AGG(S) | 0 | 0 | 0 | 0 | 0 | 0 |
| Gly (G) | GGU(G) | 31 | 0.53 | 12 | 0.35 | 19 | 0.77 |
|  | GGC(G) | 36 | 0.61 | 20 | 0.59 | 16 | 0.65 |
|  | **GGA(G)** | 84 | 1.43 | 70 | 2.06 | 14 | 0.57 |
|  | GGG(G) | 84 | 1.43 | 34 | 1 | 50 | 2.02 |

Table S6. Best partitioning schemes and models selected by PartitionFinder for phylogenetic analyses.

| **Dataset** | **Subset Partitions** | **Best Model** |
| --- | --- | --- |
| PCGRNA:  6 partitions | P1: (CO1_pos1, CO2_pos1, ND3_pos1, ATP6_pos1, CO3_pos1, CytB_pos1) | GTR+I+G |
|  | P2: (ATP6_pos2, ND1_pos2, CO3_pos2, CO1_pos2, CO2_pos2, CytB_pos2, ND3_pos2, ND4L_pos2, ND4_pos2, ND5_pos2, ND6_pos2, ATP8_pos2, ND2_pos2) | GTR+I+G |
|  | P3: (CytB_pos3, CO3_pos3, ND6_pos3, ATP8_pos3, ND2_pos3, CO1_pos3, CO2_pos3, ND3_pos3, ATP6_pos3) | HKY+I+G |
|  | P4: (tRNA_NT, ND1_pos1, ND5_pos1, ND4_pos1, ND4L_pos1, ATP8_pos1, ND6_pos1, ND2_pos1) | GTR+I+G |
|  | P5: (ND1_pos3, ND5_pos3, ND4_pos3, ND4L_pos3) | GTR+I+G |
|  | P6: (12S_NT, 16S_NT) | GTR+I+G |
| PCG12RNA:  4 partitions | P1: (CO1_pos1, CO2_pos1, ND3_pos1, ATP6_pos1, CO3_pos1, CytB_pos1) | GTR+I+G |
|  | P2: (ATP6_pos2, ND1_pos2, CO3_pos2, CO1_pos2, CO2_pos2, CytB_pos2, ND3_pos2, ND4L_pos2, ND4_pos2, ND5_pos2, ND6_pos2, ATP8_pos2, ND2_pos2) | GTR+I+G |
|  | P3: (tRNA_NT, ND1_pos1, ND5_pos1, ND4_pos1, ND4L_pos1, ATP8_pos1, ND6_pos1, ND2_pos1) | GTR+I+G |
|  | P4: (12S_NT, 16S_NT) | GTR+I+G |
| PCG:  5 partitions | P1: (CO1_pos1, CO2_pos1, ND3_pos1, ATP6_pos1, CO3_pos1, CytB_pos1) | GTR+I+G |
|  | P2: (ATP6_pos2, ND1_pos2, CO3_pos2, CO1_pos2, CO2_pos2, CytB_pos2, ND3_pos2, ND4L_pos2, ND4_pos2, ND5_pos2, ND6_pos2, ATP8_pos2, ND2_pos2) | GTR+I+G |
|  | P3: (CytB_pos3, CO3_pos3, ND6_pos3, ATP8_pos3, ND2_pos3, CO1_pos3, CO2_pos3, ND3_pos3, ATP6_pos3) | HKY+I+G |
|  | P4: (ND1_pos1, ND5_pos1, ND4_pos1, ND4L_pos1, ATP8_pos1, ND6_pos1, ND2_pos1) | GTR+I+G |
|  | P5: (ND1_pos3, ND5_pos3, ND4_pos3, ND4L_pos3) | GTR+I+G |
| PCG12:  3 partitions | P1: (CO1_pos1, CO2_pos1, ND3_pos1, ATP6_pos1, CO3_pos1, CytB_pos1) | GTR+I+G |
|  | P2: (ATP6_pos2, ND1_pos2, CO3_pos2, CO1_pos2, CO2_pos2, CytB_pos2, ND3_pos2, ND4L_pos2, ND4_pos2, ND5_pos2, ND6_pos2, ATP8_pos2, ND2_pos2) | GTR+I+G |
|  | P3: (ND1_pos1, ND5_pos1, ND4_pos1, ND4L_pos1, ATP8_pos1, ND6_pos1, ND2_pos1) | GTR+I+G |
